# Distinct radial glia subtypes regulate midbrain dopaminergic neuron development

**DOI:** 10.1038/s41593-026-02200-8

**Published:** 2026-02-16

**Authors:** Emilía Sif Ásgrímsdóttir, Luca Fusar Bassini, Ting Sun, Clàudia Puigsasllosas Pastor, Pia Rivetti di Val Cervo, Daniel Gyllborg, Kawai Lee, Christopher L. Grigsby, Baptiste Jude, Carmen Abaurre, Saiful Islam, Peter Lönnerberg, Carlos Villaescusa, Carmen Saltó, Roger A. Barker, Sten Linnarsson, Goncalo Castelo-Branco, Gioele La Manno, Enrique M. Toledo, Ernest Arenas

**Affiliations:** 1 Laboratory of Molecular Neurobiology, Department of Medical Biochemistry and Biophysics, Karolinska Institutet, Stockholm, Sweden; 2 Laboratory of Neurodevelopmental Systems Biology, EPFL, Lausanne, Switzerland; 3 Laboratory of Medical Systems Bioengineering, Department of Medical Biochemistry and Biophysics, Karolinska Institutet, Stockholm, Sweden; 4 Molecular Muscle Physiology & Pathophysiology Group, Department of Physiology & Pharmacology, Karolinska Institutet, Solna, Sweden; 5 Division of Pediatric Neurology, Department of Women’s and Children’s Health, Karolinska Institutet, Solna, Sweden; 6 John van Geest Centre for Brain Repair, Department of Clinical Neurosciences and Cambridge Stem Cell Institute, University of Cambridge, Cambridge, UK; 7 Present Address: Laboratory of Neural Stem Cells and Brain Damage, Department of Biomedical Sciences, Institute of Neurosciences, University of Barcelona, Barcelona, Spain; 8 Present Address: Italian Medicines Agency (AIFA), Rome, Italy; 9 Present Address: Department of Biochemistry and Biophysics, Stockholm University, Science for Life Laboratory, Solna, Sweden; 10 Present Address: School of Pharmacy, Queens University Belfast, Belfast, UK; 11 Present Address: Freenome, San Francisco, CA USA; 12 Present Address: Cell Therapy R&D, Novo Nordisk A/S, Måløv, Denmark; 13 Present Address: AI and Digital Innovation, Novo Nordisk Research Centre Oxford, Oxford, UK

**Keywords:** Neuronal development, Stem-cell niche, Cell fate and cell lineage, Stem-cell differentiation

## Abstract

Understanding the development of midbrain dopaminergic (mesDA) neurons is essential for advancing cell replacement therapies for Parkinson’s disease. In the developing ventral midbrain (VM), radial glia (Rgl) cells are the progenitors of mesDA neurons. However, distinct Rgl subtypes have recently been identified, and their individual roles are unclear. Here we analyze transcriptomic data from mouse and human VM Rgl to define their contributions to mesDA neuron development. We identify Rgl1 as the progenitor of the mesDA lineage, and reveal a Rgl1 transcriptional network coordinated by *BMAL1*, which we validate as a new regulator of mesDA neurogenesis. Moreover, we uncover Rgl3 as a key signaling subtype and show that factors expressed by Rgl3 promote the survival and yield of human stem cell-derived mesDA neurons. Our findings delineate distinct roles of Rgl subtypes, elucidate lineage relationships in the developing VM and uncover new factors that improve the derivation of clinically relevant human mesDA neurons.

## Abstract (web summary)

The authors find that distinct radial glia subtypes generate and support midbrain dopaminergic neurons, revealing specialized function and lineage relationships among the diverse cell types that shape dopamine neuron development.

## Main

Midbrain dopaminergic (mesDA) neurons selectively degenerate in Parkinson’s disease (PD) and understanding their development has been crucial to advancing PD cell replacement therapy^1^. mesDA neurons originate among radial glia (Rgl) in the floor plate (FP) of the ventral midbrain (VM), under the influence of two key signaling centers—the isthmic organizer at the midbrain–hindbrain boundary^2^ and the midbrain floor plate (mFP)^3^. In recent years, single-cell RNA sequencing (scRNA-seq) has revealed the cellular diversity of the developing VM and provided new insights into mesDA neuron development^4–9^. However, the specific roles of individual cell types and how their signaling and extracellular matrix (ECM) factors shape the microenvironment of the developing VM remain to be elucidated.

Rgl are transient, heterogeneous cells in the developing central nervous system that contribute to multiple aspects of neurodevelopment^10^. First described by Camilo Golgi, and later proposed to act as a glial scaffold for migrating neurons by Giuseppe Magini^11^, Rgl were eventually recognized as neural stem cells, capable of limited self-renewal and of giving rise to both neurons and glia^12^. However, their functions vary along the neuraxis, with FP Rgl generally considered non-neurogenic and influencing neurodevelopment through secreted factors^3^, whereas Rgl in the mFP appear unique in their abilities to undergo neurogenic divisions to give rise to mesDA neurons^13,14^. More recently, scRNA-seq identified three transcriptionally and spatially distinct Rgl in the developing human and mouse VM (Rgl1-Rgl3)^4,6^, but their individual contributions to mesDA neurodevelopment are still unclear.

Here we integrate bulk and single-cell transcriptomic data from the developing mouse and human VM to define the roles of Rgl subtypes in mesDA neuron development. We identify Rgl3 as a signaling cell type and demonstrate that ECM molecules expressed by Rgl3 promote survival and improve the yield of mesDA neurons derived from human embryonic stem cells (hESCs). In contrast, Rgl1 was defined by a neurogenic transcriptional network centered on the transcription factor (TF) *BMAL1*, which we uncovered as a new regulator of mesDA neurogenesis. Finally, lentiviral barcode-based single-cell lineage tracing coupled with scRNA-seq establishes Rgl1 as the shared progenitor of both mesDA neuroblasts and Rgl3, revealing how a single progenitor can give rise to both the neuronal lineage and the niche cell type that supports its development.

Together, these findings provide a functional dissection of Rgl subtypes in the developing VM, uncover lineage relationships among midbrain cell types and reveal new TFs and ligands that enhance the derivation of mesDA neurons from human stem cells.

## Results

### Transcriptomic analysis of the embryonic mouse VM during dopaminergic neuron development

**Fig. 1:**
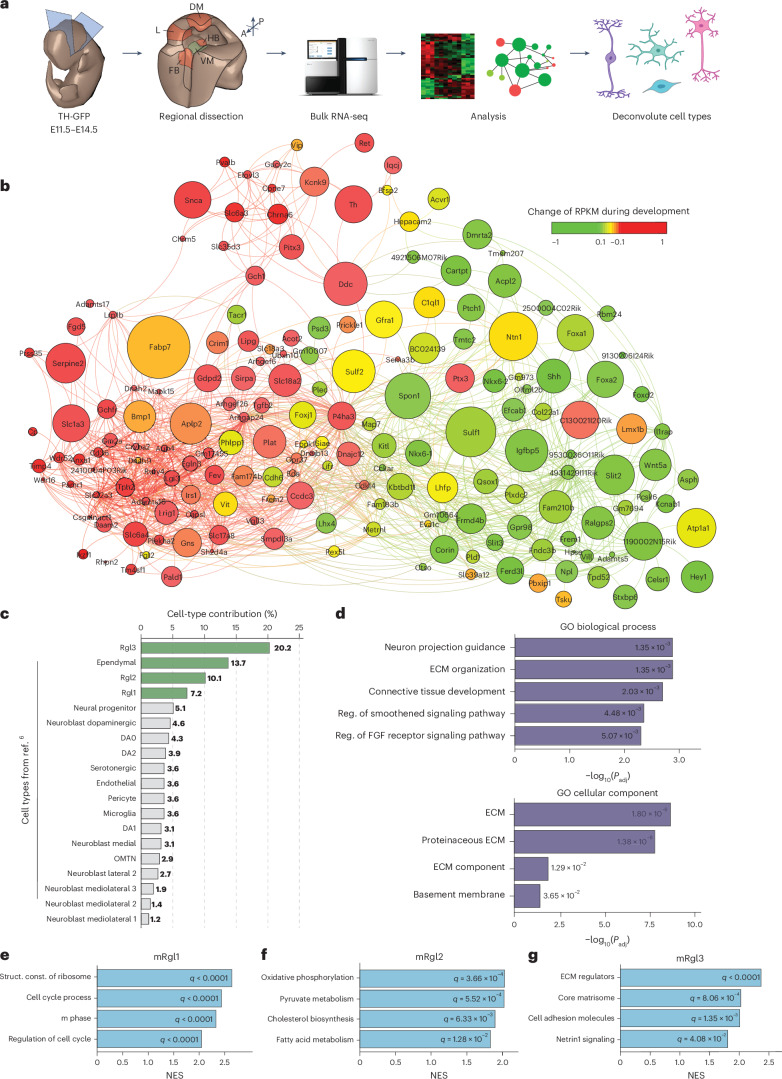
Transcriptomic profiling of the developing mouse VM identifies a dopaminergic gene coexpression module. **a**, Schematic overview of the experimental workflow. Embryonic tissue from TH-GFP mouse embryos was collected for bulk RNA-seq at E11.5, E12.5, E13.5 and E14.5. Regions sampled included the VM, DM, ventral forebrain, ventral hindbrain and alar plate (L). Bulk RNA-seq data were used to define the transcriptional network of the dopaminergic (mesDA) module. Contributions of individual VM cell types to this network were inferred using scRNA-seq data from the developing mouse VM^6^. **b**, WGCNA of the mesDA module, filtered for the top 5% interactions. Node color represents gene expression changes during development, and node size corresponds to mean expression level. **c**, Contribution of VM cell types to the transcriptional network in **b**, based on cell-type-specific expression profiles from ref. ^6^. **d**, GO term enrichment for the top four contributing cell types of the mesDA module (Rgl1–Rgl3 and ependymal cells), determined using two-sided Fisher’s exact test with Benjamini–Hochberg correction. Individual cell-type transcriptomes based on data from ref. ^6^. Top, GO biological process (GO:0008150). Bottom, GO cellular component (GO:0005575). **e**–**g**, GSEA for mRgl1 (**e**), mRgl2 (**f**) and mRgl3 (**g**). Selected top-ranking terms based on NES from the MSigDB C2 and C5 gene sets (v5.0) are shown. DM, dorsal midbrain; NES, normalized enrichment scores. Source data

**Extended Data Fig. 1:**
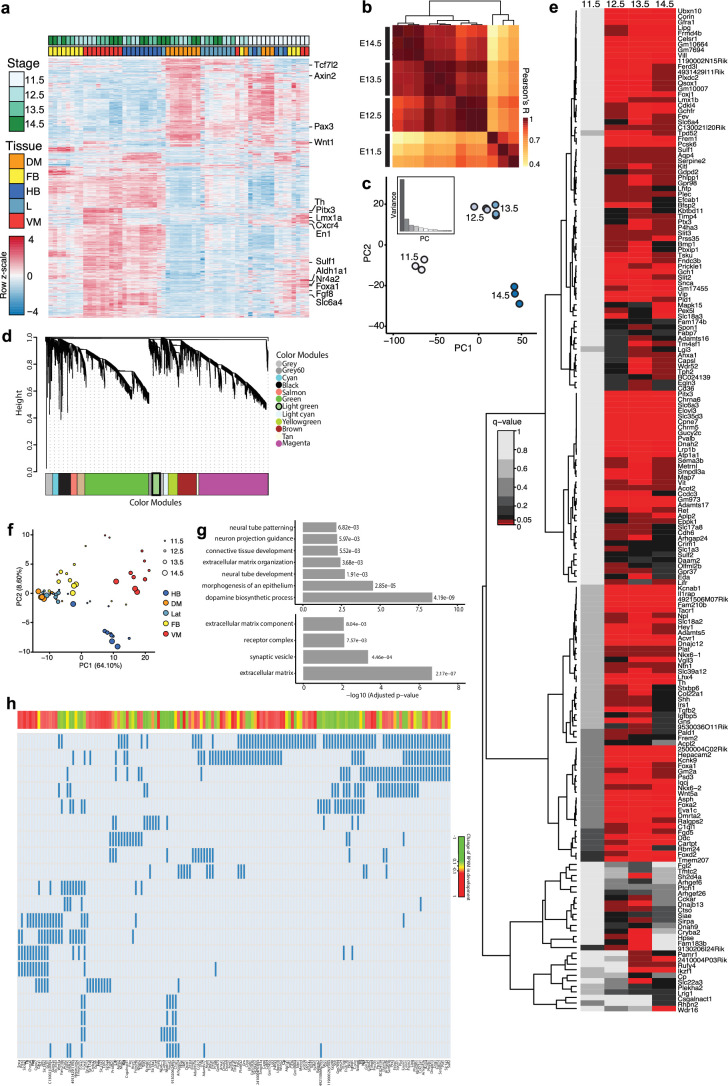
Bulk RNA-seq and weighted gene coexpression network analysis of the developing mouse VM. **a**, Heatmap representation of highly variable genes (rows) across all samples (columns) from E11.5 to E14.5 in different brain regions. Gene expression values are z-score normalized. Samples are color-coded by developmental stage and region. Selected genes associated with mesDA neuron development are labeled. **b**, Pearson’s correlation of VM samples, after filtering for top 12.5% most variable genes. **c**, Principal component analysis (PCA) of VM samples at different developmental stages. Inset histogram shows the percentage of variance explained by each principal component. **d**, Dendrogram of gene coexpression modules obtained from WGCNA. The mesDA module is shown in light green with a black border. **e**, PCA of all brain regions and developmental stages analyzed. Genes in the mesDA module were sufficient to separate the VM from the rest of the brain region samples. Samples are color-coded based on region and size represents developmental stage. **f**, Heatmap of DEGs in the mesDA module across developmental stages (E11.5–E14.5). Red scale, q < 0.05; gray scale, q > 0.05. **g**, GO analysis of genes in the mesDA module, determined using two-sided Fisher’s exact test with Benjamini–Hochberg correction. Top panel: GO terms for biological processes (GO:0008150). Bottom panel: GO terms for cellular components (GO:0005575). **h**, Identity matrix of VM cell types (rows) and genes (columns) in the mesDA module. Dark blue lines indicate genes expressed above baseline in each cell type. Color scale represents changes in expression during development. Red = increased expression, yellow = maintained, and green = decreased. Source data

To define region-specific and stage-specific transcriptional programs in the developing VM, we performed bulk RNA-seq on embryonic mouse VM, ventral hindbrain, ventral forebrain, dorsal midbrain and alar plate (L) tissue from E11.5, E12.5, E13.5 and E14.5, respectively (Fig. 1a). Multiple differentially expressed genes (DEGs) were identified in the VM across all time points (Extended Data Fig. 1a), and principal component analysis (PCA) of VM samples revealed E11.5 as the most transcriptionally divergent stage (Extended Data Fig. 1b,c). Next, we identified modules of coexpressed genes using weighted gene coexpression network analysis (WGCNA), revealing a module (light green) enriched in VM DEGs from E11.5 to E14.5 (Extended Data Fig. 1d,e and Supplementary Table 1). The top 5% interactions in this module, involving 48% of its genes, were enough to separate the VM from adjacent regions across all time points (Extended Data Fig. 1f). This refined module contained several genes associated with mesDA neuron development and was therefore designated as the mesDA module.

The mesDA module formed a network of temporally coordinated, interconnected genes (Fig. 1b) and Gene Ontology (GO) analysis of the network identified several terms relevant to midbrain development (Extended Data Fig. 1g). Notably, the ECM was among the most enriched terms, suggesting a more prominent role for the ECM in mesDA neuron development than currently recognized. To assign cell types to this network, we integrated published scRNA-seq data from the developing mouse VM^6^. Of the 181 genes in the network, 176 (97%) were assigned to at least one cell type (Extended Data Fig. 1h). Surprisingly, 51% of genes were contributed by mouse Rgl1–Rgl3 (mRgl1–mRgl3) and ependymal cells (Fig. 1c), with GO analysis linking these cell types to the ECM, neuron projection guidance and Shh/Fgf signaling (Fig. 1d).

Because mRgl1–mRgl3 are VM-specific^6^, we performed Gene Set Enrichment Analysis (GSEA) to explore their functions in VM development, revealing an enrichment in genes related to proliferation in mRgl1 (Fig. 1e), mitochondrial respiration and lipid metabolism in mRgl2 (Fig. 1f) and ECM components and signaling in mRgl3 (Fig. 1g). These results suggested a functional specialization of VM Rgl and led us to examine their individual roles in further detail.

### mRgl3 is a major signaling source in the developing mouse midbrain

**Fig. 2:**
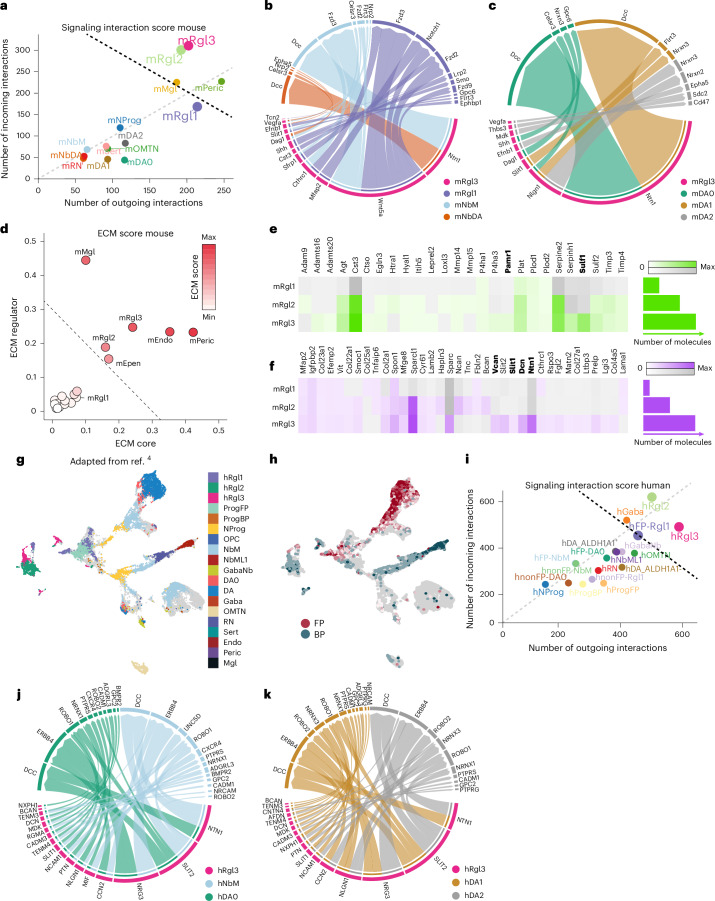
Rgl3 is a signaling cell type in the developing mouse and human VM. **a**, Scatterplot showing the number of incoming and outgoing signaling interactions for individual mouse VM cell types, as predicted by CellChat. The black dotted line represents the 95th percentile of bootstrapped total interaction scores. Gray dotted line (*x* = *y*) separates cell types with more outgoing (above) or incoming (below) interactions. Dots corresponding to Rgl cell types are enlarged for visualization purposes. **b**,**c**, Chord diagrams illustrating predicted outgoing signaling interactions of mRgl3 to cell types in the mouse mesDA lineage. mRgl3 is predicted to interact with receptors expressed by (**b**) mRgl1, mNbM and mNbDA, and (**c**) dopaminergic neurons (DA0–DA2). Chord thickness reflects interaction probability; only interactions involving >10 cells are shown. **d**, Scatterplot showing ECM scores for mouse VM cell types based on expression of ECM core components and regulators, as defined by the Matrisome project. The dotted line indicates the 99.9th percentile of bootstrapped ECM scores. **e**,**f**, Heatmaps showing expression of (**e**) ECM regulators and (**f**) ECM core components in Rgl cell types in the developing mouse VM. Color intensity reflects the Bayesian estimate of expression, and the gray scale indicates values below the significance threshold. Bar plots (right) show average total transcript levels per cell type. mRgl3-specific ECM genes are shown in bold. **g**, UMAP adapted from ref. ^4^ showing cells in the developing human VM (*FOXA2*/*FOXA1*^+^ or *TH*^+^) from weeks 5 to 14 postconception. Cell-type identity was predicted using logistic regression trained on human VM cell types from ref. ^6^. Colored cells, prediction probability >60%; gray, <60%. **h**, UMAP of human VM cells annotated based on enriched FP (*FOXA2*^+^/*LMX1A*^+^/*EN1*^+^) or BP (*NKX2.2*^*+*^, *NKX6.1*^*+*^ or *NKX6.2*^*+*^) identity. **i**, Scatterplot showing incoming and outgoing signaling interactions for human VM cell types^4^, as predicted by CellChat. mesDA lineage cell types expressing posterior midbrain markers (*LMX1A*^*+*^/*FOXA2*^*+*^/*EN1*^*+*^) were classified as FP. Dots corresponding to Rgl cell types are enlarged for visualization purposes. **j**,**k**, Chord diagrams showing predicted outgoing interactions from hRgl3 with receptors expressed by (**j**) hNbM and hDA0 and (**k**) hDA-ALDH1A1^+^ and hDA-ALDH1A1^−^. Only interactions with a communication probability >3% are shown. BP, basal plate; mNbM, mNeuroblastMedial; mNbDA, mNeuroblastDopaminergic. Source data

**Extended Data Fig. 2:**
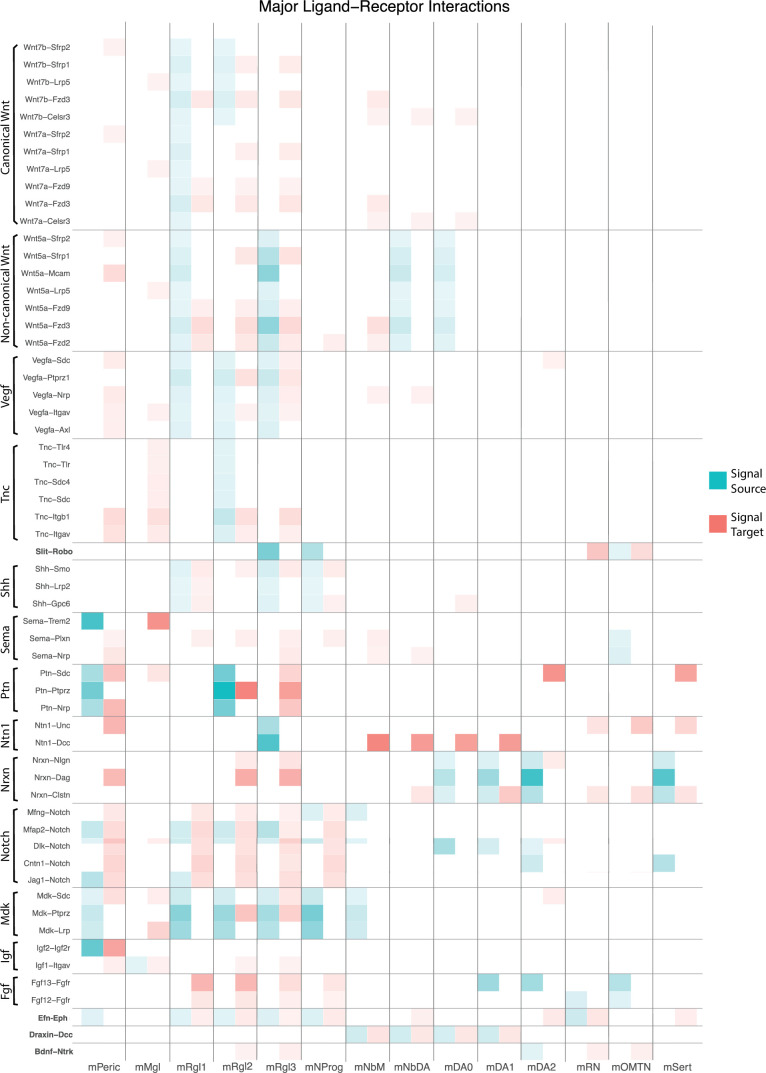
Major predicted ligand–receptor Interactions in the developing mouse VM. Heatmap showing major predicted ligand–receptor interactions in cell types in the developing mouse VM. Gene expression was filtered based on a Bayesian posterior probability threshold, and only cell types with predicted interactions are shown. Pathways are indicated on the left, with interactions grouped by ligand–receptor families. Notch: Notch1-4 and Dlk1-2. Fgf: Fgf1-3 and Fgf12-13. Slit-Robo: Slit1-3 and Robo1,2,4. Nrxn: Nrxn1-3. Ntn1: Ntn1 and Unc5a-Unc5d. Efn-Eph: Efna1,Efna2,Efna5/Efnb1,Efnb2 and Epha1-Epha5/Ephb1-Ephb3. Sema: Sema3c,Semad,Semaf/Nrp1,Nrp2 and Sema3c,Semad,Semaf,Semag/Sema4c,Sema4g/Sema5a/Sema6a,Sema6b,Sema6c/Sema7a/Plxna1-Plxna4,Plxnb1-Plxnb4. Mdk: Mdk/Sdc2,Sdc4 and Mdk/Lrp1. Ptn: Ptn/Sdc2,Sdc3,Sdc4 and Ptn/Nrp1. Tnc: Tnc/Sdc1,Sdc4 and Tnc/Tlr4. Vegfa: Vegfa/Nrp1 and Vegfa/Sdc2,Sdc3,Sdc4.

**Extended Data Fig. 3:**
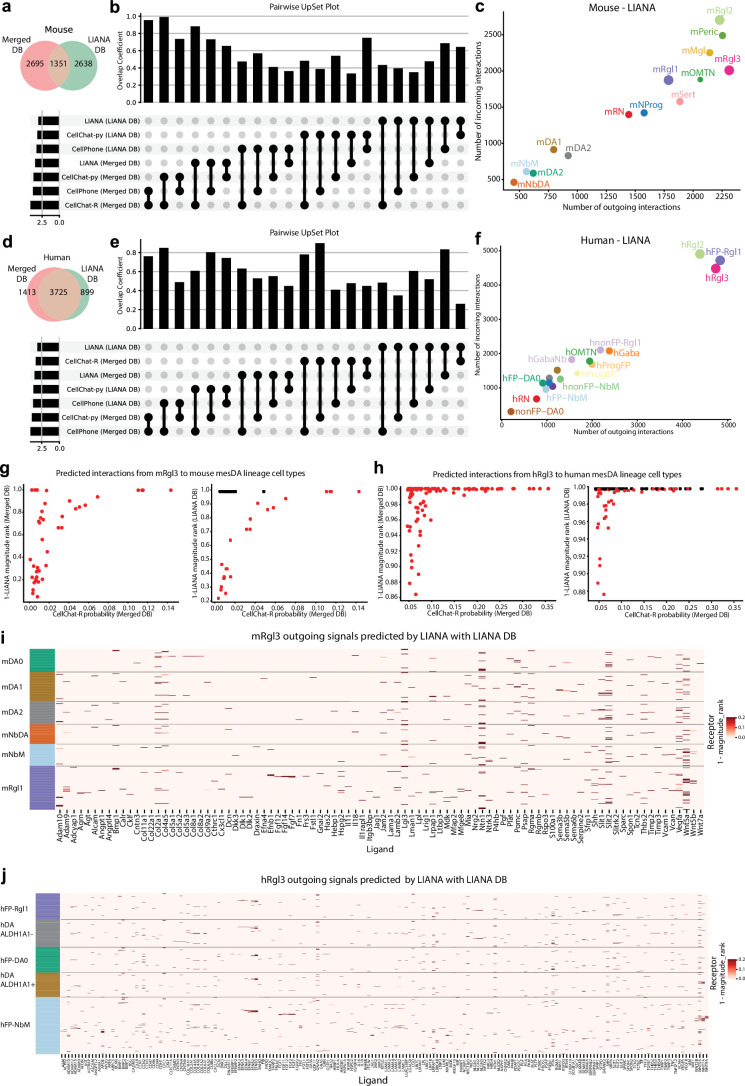
Comparison of ligand–receptor interaction predictions in the developing mouse and human VM using CellChat or LIANA. **a**,**d**, Venn diagrams showing shared and unique ligand–receptor pairs in our merged database and the LIANA database, for mouse (**a**) and human (**d**), respectively. **b**,**e**, Pairwise UpSet plots comparing predictions from our CellChat analysis of the mouse (**b**) and human (**e**) VM using different combinations of inference methods with either our merged database or the LIANA database. CellChat-R refers to original implementation of CellChat while CellChat-py refers to the Python wrapper in LIANA. **c**,**f**, Scatterplots showing the number of predicted incoming and outgoing signaling interactions per cell type in the mouse (**c**) and human (**f**) VM, as predicted by LIANA using the LIANA database. Dots corresponding to Rgl cell types are enlarged for visualization purposes. **g**,**h**, Comparison of predicted ligand–receptor interactions in the developing mouse (**g**) and human (**h**) VM. Left: predictions from CellChat using our merged database versus LIANA using our database (Spearman’s R = 0.57 for mouse, 0.30 for human). Right: predictions from CellChat using our merged database versus LIANA using the LIANA database. Black dots indicate interactions predicted by CellChat but not by LIANA (excluding black dots, Spearman’s R = 0.94 for mouse, 0.73 for human). **i**,**j**, Heatmaps showing predicted outgoing signal interactions from mouse (**i**) and human (**j**) Rgl3 to cell types of the mesDA lineage, inferred using LIANA with the LIANA database. Color intensity represents interaction strength.

To investigate the contribution of Rgl subtypes to signaling in the developing mouse VM, we constructed an extended ligand–receptor interaction database by integrating data from CellChat, CellPhoneDB and CellTalkDB, and used CellChat to infer signaling interactions from published scRNA-seq data^6^. Analysis of incoming and outgoing signaling interactions identified mRgl2/mRgl3, pericytes and microglia as major contributors to signaling, with mRgl3 having the highest number of combined interactions (Fig. 2a). Top predicted interactions across all cell types in the dataset included pathways known to regulate mesDA neuron development (Wnt, Shh, Notch and Fgf), axon guidance and synapse formation (for example, *Ntn1*-*Dcc* and *Slit*-*Robo*) and trophic factors supporting mesDA neuron survival (for example, Pleiotrophin (*Ptn*)^15^ and Midkine (*Mdk*)^16^; Extended Data Fig. 2). Focusing on outgoing interactions from mRgl3 to mesDA lineage cell types (Fig. 2b,c) revealed that Wnt, Shh and Notch signaling dominated predicted interactions of mRgl3 with mRgl1, while interactions involving the two dopaminergic neuroblast cell types (mNeuroblastMedial and mNeuroblastDopaminergic) were primarily associated with axon guidance pathways (Fig. 2b). Similarly, mRgl3 was predicted to interact with embryonic mesDA neurons (mDA0–mDA2) through axon guidance molecules (for example, *Ntn1* and *Slit1*) and synaptic adhesion proteins (for example, Nrxn-Nlgn; Fig. 2c). To ensure the robustness of our results, we compared our analysis with other cell–cell communication inference methods (Extended Data Fig. 3a,b,g) and confirmed the contribution of mRgl3 to signaling (Extended Data Fig. 3c) and its interactions with the mesDA lineage (Extended Data Fig. 3i) using LIANA^+^.

### Pericytes, microglia and mRgl3 shape the ECM landscape of the developing mouse VM

**Extended Data Fig. 4:**
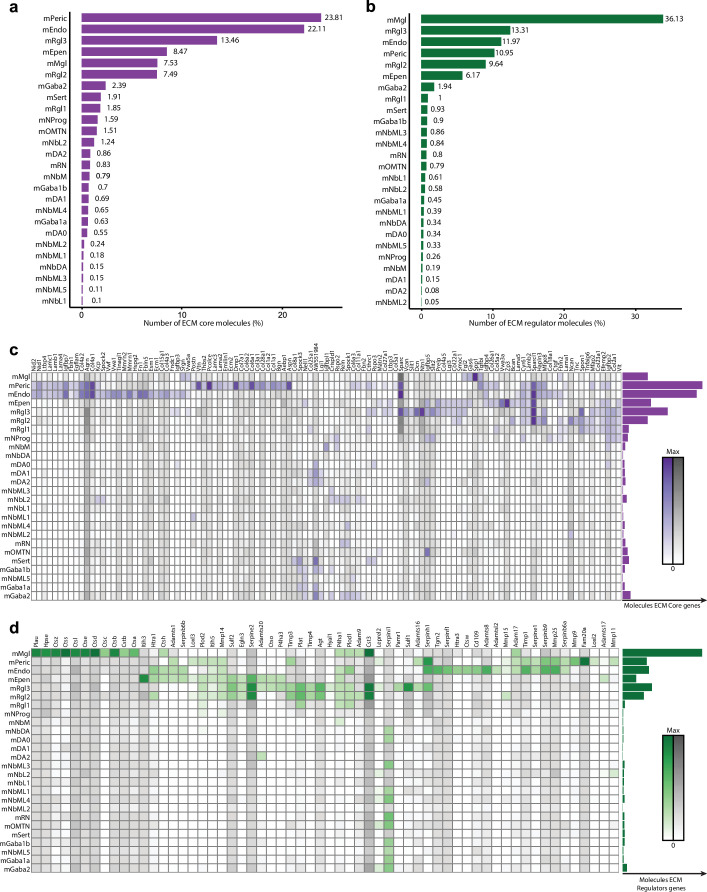
Contribution of cell types to ECM composition in the developing mouse VM. **a**,**b**, Bar plots showing the proportion of total transcripts for ECM core components (**a**) and ECM regulators (**b**) contributed by mouse VM cell types from E11.5 to E18.5. **c**,**d**, Heatmaps showing expression levels of ECM regulators (**c**) and ECM core components (**d**) across all mouse VM cell types. Color intensity is proportional to the Bayesian estimate of expression, with gray scale indicating values below the significance threshold. Bar plots on the right represent the total number of transcripts for ECM regulators/core components per cell type.

We then examined the contribution of Rgl subtypes to the ECM in the developing mouse VM. We calculated an ECM score based on Matrisome gene sets^17^, incorporating both the number of genes expressed and their expression levels, and ranked cell types by their contribution to the ECM. Six cell types (mRgl2/mRgl3, ependymal cells, endothelial cells, pericytes and microglia) exceeded a threshold set at the 99.9% quantile of the mean (Fig. 2d), accounting for 82.8% ECM core component transcripts (Extended Data Fig. 4a) and 88.2% ECM regulators in the VM (Extended Data Fig. 4b). Pericytes were the primary source of ECM core components, expressing multiple collagen and laminin genes (Extended Data Fig. 4c) essential for ECM structure and organization^18^, while microglia contributed the most ECM regulators due to their expression of multiple cathepsin proteases (Extended Data Fig. 4d), which regulate the immune response^19^.

Among Rgl subtypes, mRgl3 was identified as a main source of ECM, accounting for ~13% of both ECM regulators and core components in the VM (Extended Data Fig. 4a,b). This included ECM genes shared with other cell types, such as *Sparc* (also expressed in endothelial cells and pericytes) and *Spon1* (expressed in all Rgl), as well as mRgl3-specific ECM factors, including *Ntn1* and *Slit1* (Fig. 2e,f). Notably, CellChat predicted *Ntn1* and *Slit1* as outgoing signals from mRgl3 to cell types in the mesDA lineage (Fig. 2b,c), and these factors are known to influence mesDA axonal development and guidance^20,21^. Together, these findings identify mRgl3 as an important signaling center in the developing mouse VM, contributing both ligands and ECM factors that interact with, and may influence, the development of the mesDA lineage.

### hRgl3 mediates signaling to the mesDA lineage in the developing human VM

**Extended Data Fig. 5:**
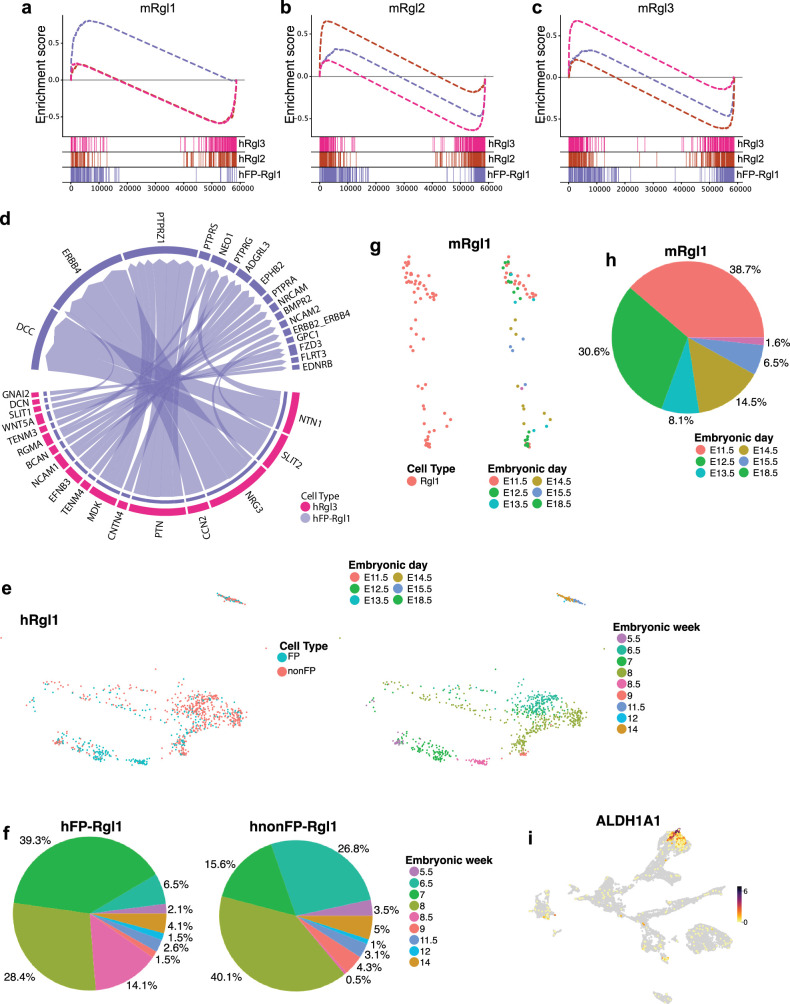
Comparison of mouse and human Rgl cell types and characterization of Rgl1 used for CellChat analysis. **a**–**c**, Gene set enrichment analysis comparing human hFP-Rgl1, hRgl2, and hRgl3 transcriptomes with genes uniquely expressed in mouse Rgl1 (**a**), mRgl2 (**b**), and mRgl3 (**c**). **a**, hFP-Rgl1: NES = 1.369, FDR < 0.001. hRgl2: NES = −1.115, FDR < 0.001. hRgl3: NES = −1.115, FDR < 0.001. **b**, hFP-Rgl1: NES = −1.171, FDR = 0.172. hRgl2: NES = 1.524, FDR < 0.001. hRgl3: −1.300, FDR = 0.035. **c**, hFP-Rgl1: NES = -1.159, FDR = 0.171. hRgl2: NES = −1.330, FDR = 0.018. hRgl3: NES = 1.578, FDR < 0.001. **d**, Chord diagram showing predicted outgoing interactions from hRgl3 with receptors expressed by hFP-Rgl1. Interactions with a communication probability of >3% are shown. **e**, UMAP of hRgl1 cells used for CellChat analysis, stratified by midbrain FP marker expression (FOXA2^+^/LMX1A^+^/EN1^+^) and colored by embryonic week. **f**, Pie chart showing distribution of hFP-Rgl1 and hnonFP-Rgl1 cells used for CellChat analysis based on embryonic week. **g**, UMAP of mouse Rgl1 (mRgl1) cells used for CellChat analysis colored by embryonic day. **h**, Pie chart showing distribution of mRgl1 cells used for CellChat analysis based on embryonic day. **i**, UMAP visualization of *ALDH1A1* expression in the developing human VM, based on data from ref. ^4^.

Having identified mRgl3 as a signaling center in the developing mouse VM, we then investigated whether hRgl3 has a similar role in the human VM. We analyzed a published scRNA-seq atlas of the developing human VM, which spans the period of mesDA neurogenesis (5–14 weeks postconception)^4^. All three Rgl subtypes (hRgl1–hRgl3) were detected in the human VM (Fig. 2g), and GSEA confirmed their transcriptional similarity to mRgl1–mRgl3 (Extended Data Fig. 5a–c).

Human VM cells segregated based on their expression of basal plate (*NKX6-1*, *NKX6-2* or *NKX2-2*^+^) or FP markers (*FOXA2*^*+*^/*LMX1A*^*+*^/*EN1*^*+*^; Fig. 2h), with hRgl1 detected in the FP, hRgl2 in the basal plate and hRgl3 in both regions. To focus on signaling in the midbrain FP (mFP), the region giving rise to mesDA neurons, we stratified cells of the human mesDA lineage based on their classification as FP (*LMX1A*^*+*^/*FOXA2*^*+*^/*EN1*^+^) or non-FP before inferring signaling interactions.

hRgl3 had the highest number of outgoing interactions among VM cell types (Fig. 2i), consistent with a major role in intercellular signaling during human VM development. hRgl3 was predicted to signal to all mesDA lineage cell types, although these interactions were surprisingly more diverse but less cell-type-specific than in the mouse (Fig. 2j,k). Most predicted hRgl3 ligands were associated with axon guidance, cell migration or neuronal survival (Fig. 2j,k and Extended Data Fig. 5d). The most frequently predicted were *NTN1* and *SLIT2*, but interacting with different receptors in premitotic and postmitotic cells, for example, *NTN1*–*NEO* in hFP-Rgl1 (Extended Data Fig. 5d) and *NTN1–UNC5D* in hFP–NbM and hFP–DA0 (Fig. 2j), suggesting cell-type-specific signal transduction. Other signaling interactions to hFP-Rgl1 conserved across species included *WNT5A*, while *SHH* and *NOTCH* were not detected in the human dataset (Extended Data Fig. 5d). These differences may reflect species-specific signaling or differences in timing of Rgl1 capture (weeks 7–8 in human versus E11.5–12.5 in mouse Rgl1, approximately weeks 6–7 in human^22^ (Extended Data Fig. 5e–g)).

hRgl3 was also predicted to signal to postmitotic mesDA lineage cell types, including interactions with hFP-NbM mediated by *MIF–CXCR4* and *NLGN–NRXN*/*CADM*, which regulate mesDA neuroblast migration^23^ and synapse formation^24^, respectively (Fig. 2j). Additionally, hRgl3 was predicted to signal to both ALDH1A1⁺ and ALDH1A1^−^ embryonic mesDA neuron subtypes (Extended Data Fig. 5i), with subtype-specific interactions such as *TENM3*/*4-ADGRL3* selectively predicted for ALDH1A1^−^ neurons (Fig. 2k), suggesting possible subtype differences in synapse organization and axon guidance^25^.

To validate the robustness of these predictions, we compared results across additional cell–cell communication inference methods (Extended Data Fig. 3d,e,h), and confirmed the contribution of hRgl3 to signaling (Extended Data Fig. 3f) and its interactions with the mesDA lineage (Extended Data Fig. 3j) using LIANA^+^.

Together, these results identify Rgl3 as a signaling center in the human and mouse developing VM and suggest that Rgl3-specific factors may interact with the mesDA lineage in a pathway-specific and cell-type-specific manner. These findings prompted us to test whether Rgl3-expressed factors regulate human mesDA neuron development in vitro.

### Rgl3-expressed ligands regulate human mesDA progenitor proliferation and survival

**Extended Data Fig. 6:**
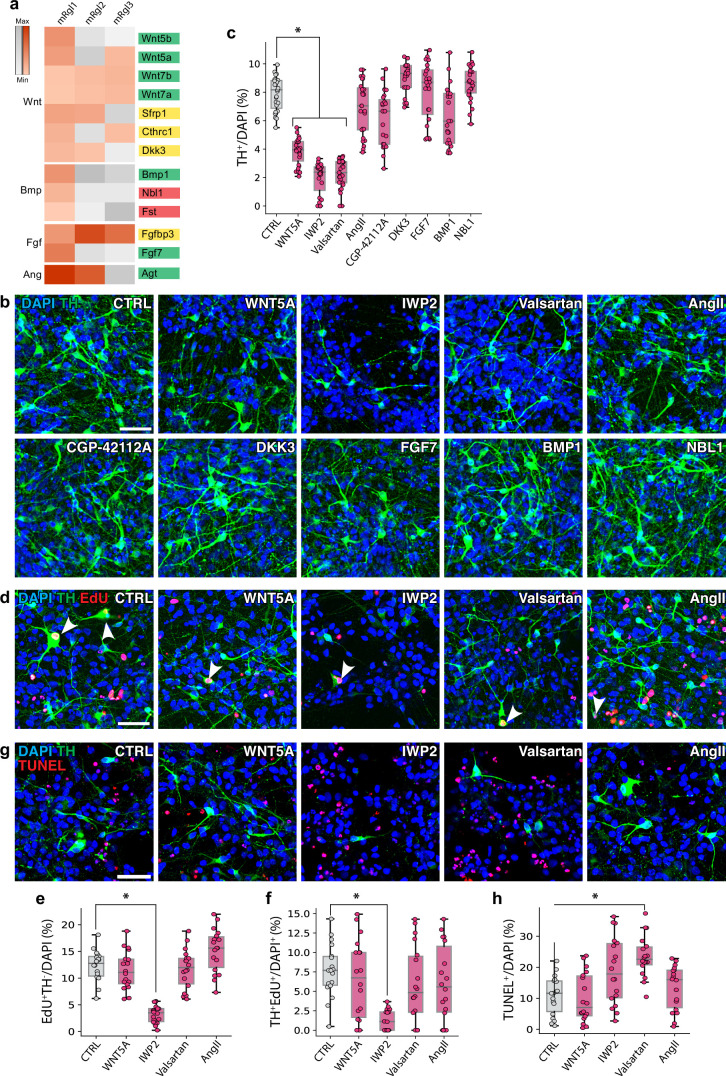
Rgl3-specific ligands influence different aspects of mesDA neuron development. **a**, Heatmap showing selected ligands expressed by Rgl cell types in the developing mouse VM. Ligands are color-coded by activity: green for activation, red for inhibition, and yellow for context-dependent modulation. Pathways are indicated on the left. Color intensity is proportional to the Bayesian estimate of expression, with gray scale indicating values below the significance threshold. **b**, Representative immunofluorescence images of TH^+^ neurons on day 35 following treatment with indicated ligands or small molecules. Scale bar, 100 µm. **c**, Quantification of TH^+^ neurons (TH^+^/DAPI^+^) across conditions. CTRL: 7.91 ± 0.39%; WNT5A: 3.83 ± 0.36% (p = 0.049); IWP2: 1.96 ± 0.05% (p = 0.044); Valsartan: 2.26 ± 0.07% (p = 0.044); AngII: 6.85 ± 0.72%; CGP-42112A: 6.24 ± 0.60%; DKK3: 9.0 ± 0.15%; FGF7: 8.07 ± 0.45%; BMP1: 6.28 ± 0.17%; NBL1: 8.63 ± 0.19%; mean ± s.e.m. **d**,**e**, Representative immunofluorescence images used to quantify change in mDA neurogenesis (TH^−^EdU^+^/DAPI^+^ arrowheads) following treatment with selected ligands and small molecules. CTRL: 7.67 ± 1.13%; WNT5A: 6.51 ± 2.83%; IWP2: 1.31 ± 0.54% (p = 0.038); Valsartan: 5.89 ± 2.50%; AngII: 6.06 ± 2.91%; mean ± s.e.m. Scale bar, 100 µm. **d**,**f**, Representative immunofluorescence images used to quantify change in proliferation (TH^+^EdU^+^/DAPI^+^ cells) following treatment with selected ligands and small molecules. CTRL: 12.3 ± 0.75%; WNT5A: 11.5 ± 1.12%; IWP2: 3.22 ± 0.18% (p = 0.037); Valsartan: 11.7 ± 1.61%; AngII: 15.2% ± 0.62%; mean ± s.e.m. Scale bar, 100 µm. **g**,**h**, Representative immunofluorescence images used to quantify reduced cell death (TUNEL^+^/DAPI^+^ cells) following treatment with selected ligands and small molecules. CTRL: 10.8 ± 3.17%; WNT5A: 10.1 ± 5.04%; IWP2: 18.9 ± 5.41%; Valsartan: 23.5 ± 2.67% (p = 0.044); AngII: 13.4 ± 4.00%; mean ± s.e.m. Scale bar, 100 µm. Box plots (**c**,**e**,**f**,**h**) display interquartile range (IQR), median (indicated by line), and whiskers (1.5× IQR) with individual data points shown as a scatterplot. Asterisks indicate statistical significance (*p < 0.05), determined using two-tailed Student’s t-test with Bonferroni–Holm correction from n = 3 independent differentiations, 2 wells per condition. Source data

To assess the role of Rgl3-specific ligands in human mesDA neuron development, we curated candidate factors (Extended Data Fig. 6a) based on predictions from CellChat and LIANA^+^. We selected ligands from the Wnt (WNT5A, IWP2, DKK3), Bmp (BMP1, NBL1) and angiotensin II (AngII, Valsartan, CGP-42112A) pathways for testing in hESC-derived mesDA cultures.

WA09 hESCs were differentiated toward the mesDA lineage and treated with candidate ligands or pathway modulators from days 22 to 35 of differentiation, coinciding with the emergence of Rgl3 and the onset of neuroblast differentiation in the culture^26^. While quantification of tyrosine hydroxylase-positive (TH^+^) mesDA neurons on day 35 revealed no significant effect for some ligands, modulation of the Wnt signaling pathway or inhibition of the angiotensin receptor AGTR1 resulted in a significant decrease in TH^+^ neurons. Specifically, inhibition of Wnt secretion with IWP2 reduced TH^+^ neuron yield by 75%, and the treatment with the noncanonical Wnt activator WNT5A by 52%, while AGTR1 inhibition with Valsartan decreased TH⁺ neurons by 71% compared to control (Extended Data Fig. 6b,c). Notably, *AGTR1* is expressed in a human mesDA neuron subtype recently associated with PD^27^, but its role in mesDA neuron development has not been examined.

To investigate the mechanism underlying this reduction in TH^+^ neurons, we administered a 4-h EdU pulse on day 24 to label proliferating progenitors. Notably, IWP2 treatment significantly reduced both mesDA neurogenesis (83%) and overall proliferation (74%) compared to control (Extended Data Fig. 6d–f), underscoring the essential role of Wnt signaling in mesDA progenitor proliferation and neurogenesis. Conversely, TUNEL staining revealed that Valsartan significantly increased cell death (181% increase in TUNEL^+^ apoptotic nuclei; Extended Data Fig. 6g,h), which together with the significant reduction in TH^+^ neurons (Extended Data Fig. 6b,c) suggests a new role for AGTR1 in supporting the survival of developing mesDA neurons.

### Rgl3-specific ECM proteins enhance survival and the yield of human TH^+^ neurons

**Fig. 3:**
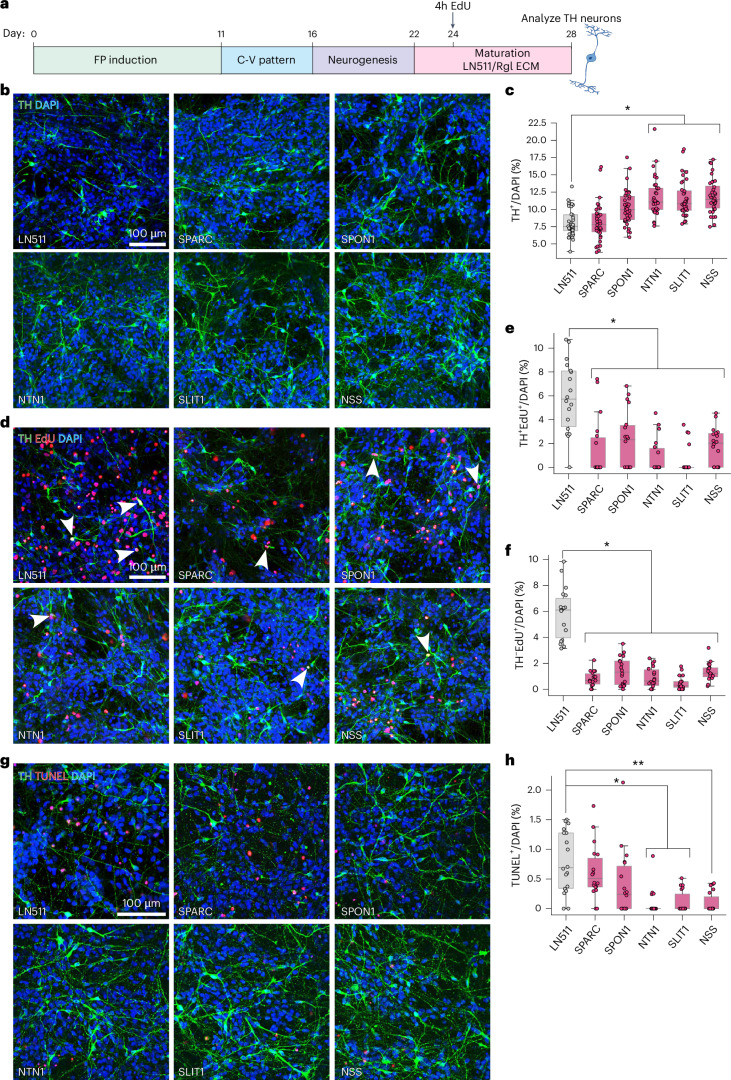
hRgl3 ECM proteins enhance survival and yield of dopaminergic neurons derived from human stem cells. **a**, Schematic of the differentiation protocol used to generate mesDA neurons from hESCs. Cells were cultured on plates coated with Laminin 511 (LN511) or individual Rgl3 ECM proteins from days 22 to 28. Cells were pulsed with 10 μM EdU pulse for 4 h on day 24, and analyzed on day 28. **b**, Representative immunofluorescence images of TH⁺ neurons at day 28 following the treatment with ECM proteins. NSS = NTN1 + SLIT1 + SPON1. LN511 was used as control. **c**, Quantification of TH⁺ neurons (TH⁺/DAPI⁺) across conditions. LN511 = 8.06 ± 0.53%; SPARC = 8.15 ± 0.68%; SPON1 = 10.3 ± 0.45%; NTN1 = 11.78 ± 0.78% (*P* = 0.024); SLIT1 = 11.5 ± 0.55% (*P* = 0.041); NSS = 11.9 ± 0.51% (*P* = 0.041); mean ± s.e.m. **d**,**e**, Representative images (**d**) and quantification (**e**) of mesDA neurogenesis (TH⁺EdU⁺/DAPI⁺, arrowheads) following the treatment with Rgl3 ECM proteins. LN511 = 5.88 ± 0.61%; SPARC = 1.50 ± 0.72% (*P* = 0.011); SPON1 = 2.26 ± 0.98% (*P* = 0.048); NTN1 = 0.91 ± 0.48% (*P* = 0.012); SLIT1 = 0.63 ± 0.32% (*P* = 0.015); NSS = 1.81 ± 0.11% (*P* = 0.031); mean ± s.e.m. **d**,**f**, Representative images (**d**) and quantification (**f**) of proliferation (TH^−^EdU⁺/DAPI⁺) following the treatment with Rgl3 ECM proteins. LN511 = 5.85 ± 0.50%; SPARC = 0.82 ± 0.28% (*P* = 0.013); SPON1 = 1.40 ± 0.57% (*P* = 0.019); NTN1 = 0.89 ± 0.39% (*P* = 0.014); SLIT1 = 0.48 ± 0.30% (*P* = 0.013); NSS = 1.35 ± 0.39% (*P* = 0.019); mean ± s.e.m. **g**,**h**, Representative images (**g**) and quantification (**h**) of cell death (TUNEL⁺/DAPI⁺) following the treatment with Rgl3 ECM proteins. LN511 = 0.79 ± 0.04%; SPARC = 0.62 ± 0.08%; SPON1 = 0.41 ± 0.23%; NTN1 = 0.09 ± 0.07% (*P* = 0.016); SLIT1 = 0.11 ± 0.08% (*P* = 0.016); NSS = 0.10 ± 0.02% (*P* = 0.005); mean ± s.e.m. Box plots (**c**,**e**,**f**,**h**) display IQR, median (line) and whiskers (1.5× IQR), with individual data points overlaid from *n* = 6 (**c**) or *n* = 3 (**e**,**f**,**h**) independent differentiations, 2 wells per condition (**c**,**e**,**f**,**h**). Asterisks indicate statistical significance (*P < 0.05; **P < 0.01), determined by two-tailed Student’s *t* test with Bonferroni–Holm correction. IQR, interquartile range. Source data

We then examined the effects of Rgl ECM proteins on mesDA neuron development, selecting two ECM proteins unique to Rgl3 (Ntn1 and Slit1), one common to all Rgl (Spon1), and one expressed in Rgl3, pericytes and endothelial cells (Sparc) for testing in hESC-derived mesDA neurons. Cells were cultured from days 22 to 28 on either laminin 511 (LN511), which promotes mesDA neuron differentiation and survival^28^ and serves as our standard baseline, or on the selected Rgl ECM proteins (Fig. 3a).

While SPARC and SPON1 did not significantly affect TH⁺ neuron yield compared to LN511 (Fig. 3b,c), both Rgl3-specific proteins NTN1 and SLIT1 significantly increased the proportion of TH^+^ neurons (by 46% and 43%, respectively). Notably, combining NTN1 and SLIT1 with SPON1 (NSS) did not further increase TH^+^ neuron yield, suggesting that these proteins share a similar mechanism of action. To explore this, we assessed progenitor proliferation and neurogenesis at day 24 with EdU labeling. All ECM proteins tested significantly reduced the proportion of proliferating progenitors (EdU^+^/DAPI^+^) and neurogenic cells (EdU^+^TH^+^/DAPI^+^) relative to LN511 (Fig. 3d–f). However, as this reduction did not correspond to a decrease in TH^+^ neurons, these findings suggest that all the tested ECM proteins promote neuronal maturation in vitro, rather than impairing neurogenesis.

To investigate the specific effects of NTN1 and SLIT1 on TH^+^ neuron yield, we then examined cell survival. Both proteins significantly reduced apoptosis at day 28, as measured by a decrease in TUNEL^+^ nuclei (by 89% and 86%, respectively), while SPARC and SPON1 had no effect compared to LN511 (Fig. 3g,h). These findings identify a new role for the Rgl3-specific ECM proteins NTN1 and SLIT1 in promoting cell survival, thereby increasing the yield of TH^+^ neurons derived from hESCs.

### TF networks define the subtype identity of mRgl

**Fig. 4:**
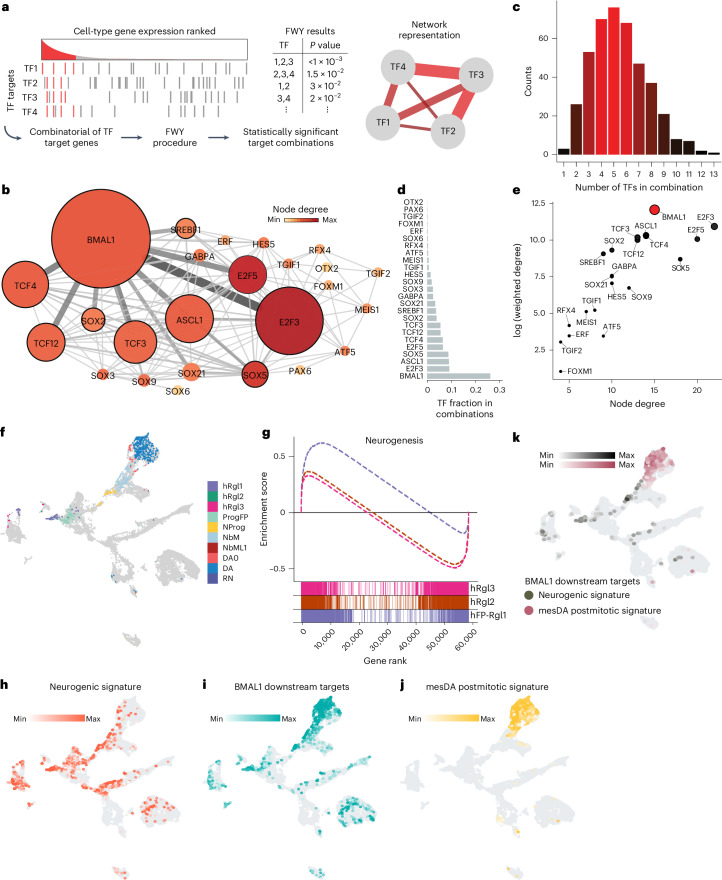
Transcriptomic analysis identifies Rgl1 as a neurogenic Rgl subtype in the developing mouse and human VM. **a**, Schematic illustrating the combinatorial analysis of TF target genes used to construct cell-type-specific transcriptional networks. Enriched TFs are identified, and their targets are ranked by expression level (left). Genes coregulated by TF1–TF4 and expressed above a defined threshold are considered enriched targets (red lines). Statistically significant TF combinations are identified using the FWY permutation procedure (middle). The resulting TF network (right) connects TFs with overlapping target genes, with edge thickness reflecting interaction strength. **b**, Mouse Rgl1 transcriptional network. Node color represents the number of TF connections (node degree), and node size reflects connection strength (weighted degree). Core nodes (high weighted degrees) are outlined in black. Edge width and color intensity reflect TF pair interaction scores. **c**, Histogram showing the number of TFs involved in significant combinations in the mRgl1 network. **d**, Bar plot showing the frequency of individual TFs across significant TF combinations. **e**, Scatterplot comparing node degree (connectivity) and weighted degree (interaction strength) for TFs in the mRgl1 network. Weighted degree is shown on a logarithmic scale. Node size and color intensity reflect weighted degree. **f**, UMAP of human VM cells is adapted from ref. ^4^, with cells expressing a posterior midbrain FP signature (LMX1A⁺/FOXA2⁺/EN1⁺) highlighted. **g**, GSEA comparing transcriptomes of FP-hRgl1, hRgl2 and hRgl3 against the human neurogenesis gene set (GO:0022008). FP hRgl1 (FP-hRgl1) was isolated based on expression of LMX1A⁺/FOXA2⁺/EN1⁺. FP-hRgl1—NES = 1.074, FDR = 0.068; hRgl2—NES = −1.072, FDR = 0.198; hRgl3—NES = –1.107, FDR = 0.116. Expression of neurogenic and postmitotic mesDA signatures in the developing human VM (**h**–**j**). **h**, UMAP showing coexpression of ≥3 TFs from the mouse Rgl1 network (BMAL1, E2F3, E2F5, ASCL1, SOX5) or NEUROG2. **i**, Expression of ≥5 BMAL1 downstream targets (BMP7, DVL2, NEUROD1, GSK3B, TCF12, NR4A2, HDAC2, IGF1R). **j**, Expression of all four mesDA postmitotic markers (EN1, NR4A2, PITX3, TH). Color intensity reflects expression level. **k**, Coexpression of BMAL1 target genes with either the neurogenic (**h**) or mesDA postmitotic (**j**) signatures. Color intensity reflects expression level. Source data

**Extended Data Fig. 7:**
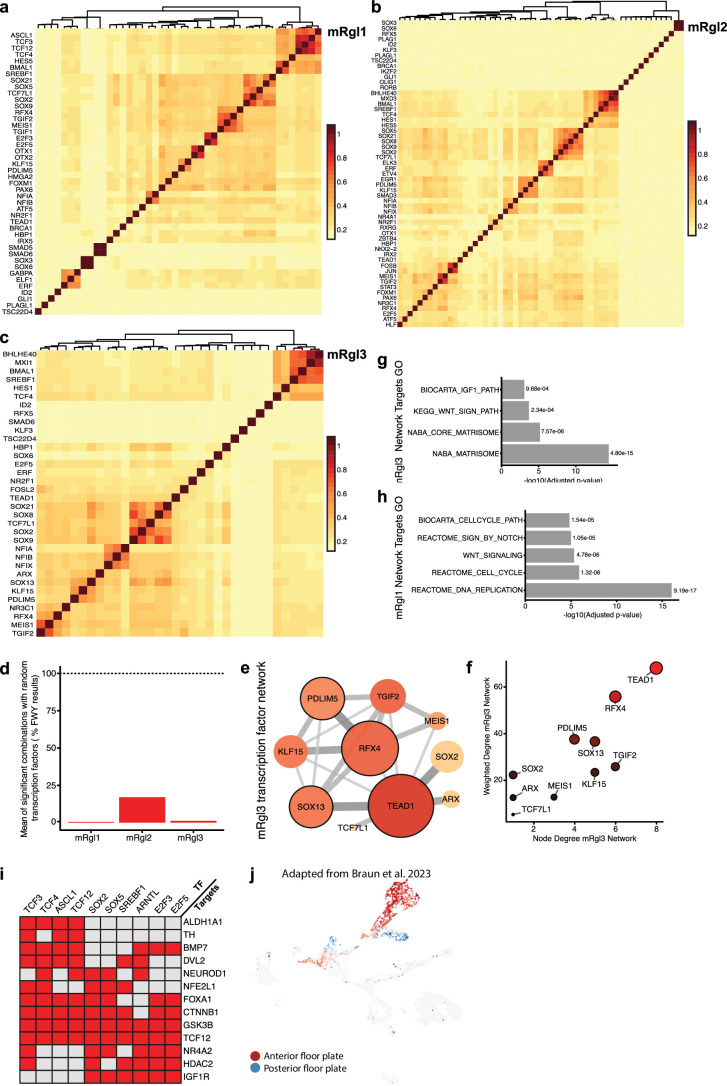
Transcription factor networks in mouse radial glia cell types. **a**–**c**, Clustering of TFs expressed in mRgl1 (**a**), mRgl2 (**b**), or mRgl3 (**c**) based on Jaccard index of shared target genes. **d**, Percentage of significant FWY combinations obtained by randomly selecting TFs from MSigDB while keeping mRgl1-3 transcriptomes constant. **e**, mRgl3 transcriptional network. Node color represents the number of TF connections (node degree), and node size reflects connection strength (weighted degree). Core nodes (high weighted degrees) are outlined in black. Edge width and color intensity reflect TF pair interaction scores. **f**, Scatterplot comparing TF node degree (connectivity) and weighted node degree (interaction strength) in mRgl3 network. Node size and color intensity correspond to weighted degree. **g**,**h**, GO analysis of target genes regulated by core TFs in the mRgl3 (**g**) and mRgl1 (**h**) TF networks, using MSigDB gene set C2 canonical pathways v5.0 and two-sided Fisher’s exact test with Benjamini–Hochberg correction. **i**, Selected target genes regulated by core TFs in the mRgl1 TF network. Target genes identified based on ref. ^74^. **j**, UMAP adapted from ref. ^4^ showing cells in the developing human VM annotated by enriched midbrain floor plate (*FOXA2*^+^/*LMX1A*^+^/*EN1*^+^) or anterior floor plate (*FOXA2*^+^/*LMX1A*^+^/*PITX2*^*+*^) identity. FWY, Fast-Westfall-Young. Source data

To further investigate the regulatory mechanisms governing mRgl subtype identity, we examined the TF networks regulating their distinct transcriptional profiles. We first identified TFs expressed by each Rgl subtype and clustered them based on shared target genes (Extended Data Fig. 7a–c). To define functionally relevant TF combinations, we used a fast Westfall–Young (FWY) permutation test to identify statistically significant TF combinations with common upregulated target genes^29^ (Fig. 4a). Notably, FWY analysis using randomly selected TFs from MSigDB while keeping the Rgl gene expression profiles constant yielded fewer significant TF combinations, confirming the specificity of the analysis (Extended Data Fig. 7d).

Analysis of mRgl3 revealed 15 enriched TF combinations (Source data Extended Data Fig. 7e), forming a network centered on *Tead1* (Extended Data Fig. 7e). GO analysis of the target genes regulated by this network revealed terms associated with the ECM, as well as Wnt and Igf signaling (Extended Data Fig. 7g), which influence mesDA neuron development and survival, respectively^30,31^.

Conversely, analysis of mRgl2 yielded only a single significant TF pair, *Pax6* and *Tcf7l1*, which was previously reported to maintain neural progenitors in an undifferentiated state^32,33^. GO analysis of their target genes revealed terms associated with forebrain (*P* = 3.12 × 10^−45^), hindbrain (*P* = 7.88 × 10^−8^) and midbrain (*P* = 5.89 × 10^−5^) development. Because Rgl2 is not located in the mFP, these findings suggest that it may perform a generic role in neural progenitor maintenance that is unlikely to directly contribute to mesDA neuron development.

### A *Bmal1*-centered transcriptional network coordinates mesDA neurogenesis in mRgl1

Analysis of mRgl1 revealed two Sox and pro-neural basic helix-loop-helix TF clusters with shared target genes (Extended Data Fig. 7a), also observed in mRgl2/mRgl3. We constructed a weighted network based on pairwise TF interaction scores, incorporating both frequency and *P* value (Fig. 4b). On average, each combination included 5.5 TFs (Fig. 4c), with *Bmal1*, *E2f3, Ascl1*, *Sox5* and *E2f5* occurring most frequently (Fig. 4d). While *E2f5* and *E2f3* had the most interaction partners (node degree), their interactions were less frequent and less significant. When node degrees were weighted for interaction score, *Bmal1* emerged as the central TF in the network (Fig. 4e), followed by *E2f3*/*E2f5, Ascl1, Sox5*/*Sox*2*, Tcf4*/*Tcf12*/*Tcf3* and *Srebf1*, suggesting that a basic helix-loop-helix neurogenic cluster predominantly defines the transcriptional state of mRgl1.

Several TFs in this network are known to regulate mesDA neurogenesis, including *Ascl1* (together with *Neurog2* (ref. ^34^), *Srebf1*(ref. ^35^) and *Tcf3*/*Tcf4* (ref. ^36^)). GO analysis of the predicted target genes regulated by the mRgl1 network revealed several terms essential for mesDA neurogenesis, including cell cycle regulation, Wnt and Notch signaling^1,37^ (Extended Data Fig. 7h). Additionally, several putative network target genes are involved in mesDA neuron development, including *Foxa1*, *Bmp7* (ref. ^38^), *Nfe2l1* (ref. ^39^), *Nr4a2* (ref. ^40^) and Wnt signaling components (for example, *Dvl2*, *Ctnnb1* and *Gsk3b*^30^). We also identified neurogenesis regulators (for example, *Neurod1* (ref. ^41^), *Hdac2* (ref. ^42^) and *Tcf12* (ref. ^43^)) and markers of mature mesDA neurons (for example, *Th* and *Aldh1a1* (ref. ^1^); Extended Data Fig. 7i).

These results suggest that the mRgl1 TF network coordinates mesDA neurogenesis by regulating progenitor proliferation, enabling mRgl1 to respond to Wnt and Shh signaling, and promoting differentiation into postmitotic mesDA neurons.

### hRgl1 is a neurogenic Rgl subtype in the developing human VM

Building on our identification of a neurogenic transcriptional network in mRgl1, we then investigated whether hRgl1 has a similar role in the developing human VM. scRNA-seq previously identified two clusters of hRgl1 in the developing human VM^4^, one expressing posterior mFP markers (*FOXA2*^*+*^/*LMX1A*^*+*^/*EN1*^*+*^) and another expressing the anterior marker *PITX2* (ref. ^4^), associated with the subthalamic nucleus^8^ (Extended Data Fig. 7i). After isolating hFP-Rgl1 based on mFP marker expression (Fig. 4f), GSEA confirmed hFP-Rgl1 as the primary neurogenic Rgl in the human VM (Fig. 4g) and identified genes from the mRgl1 network, such as *ASCL1*, *TCF3*/*TCF4*/*TCF12* and *SOX5*, that may also contribute to neurogenesis in hFP-Rgl1. Additionally, new neurogenesis-related genes previously shown to enrich for mesDA progenitors, including *TPBG* and *PTPRO*,^44,45^ were enriched in hFP-Rgl1.

To examine the presence of the mRgl1 transcriptional program in hFP-Rgl1, we analyzed the expression of a neurogenic signature comprising the five most significant TFs from the mRgl1 network, along with *NEUROG2* (ref. ^34^), as well as the expression of *BMAL1* target genes in the developing human VM (Extended Data Fig. 7j). Two cell types in the mesDA lineage expressed both signatures—hFP-Rgl1 and hNProg (Fig. 4h,i)—consistent with neurogenesis being activated along the hFP-Rgl1-NProg axis. Furthermore, *BMAL1* target genes were also expressed in embryonic mesDA neurons (*EN1*^*+*^/*NR4A2*^*+*^/*PITX3*^*+*^/*TH*^*+*^; Fig. 4j), suggesting that a neurogenic program involving *BMAL1* is initiated in hFP-Rgl1, maintained in hNProg and culminated in mesDA neurons (Fig. 4k).

### *BMAL1* regulates proliferation and the timing of neurogenesis in human mesDA progenitors

**Extended Data Fig. 8:**
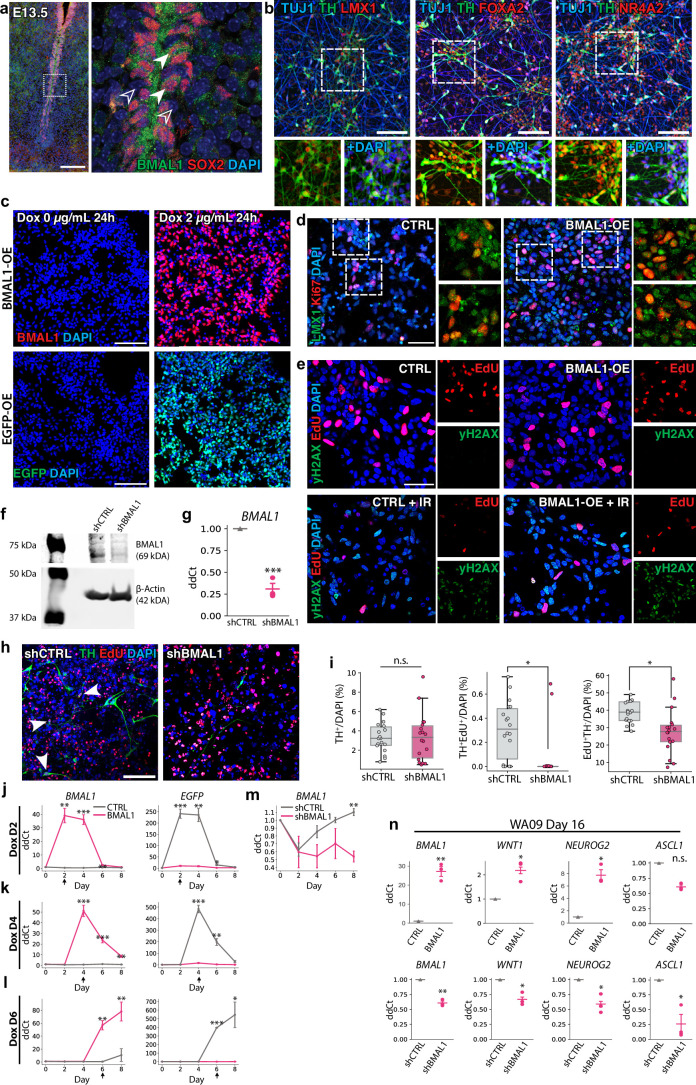
Validation and functional analysis of BMAL1 hLT-NES and hESCs. **a**, BMAL1 expression in the developing mouse VM at E13.5. Image to the right shows magnified image from white box on the left. Solid arrowheads indicate SOX2^+^BMAL1^+^ cells, while empty arrowheads indicate SOX2^−^BMAL1^−^ cells. Scale bar, 100 µm. **b**, Representative immunofluorescence images of LMX1A^+^TH^+^, FOXA2^+^TH^+^, and NR4A2^+^TH^+^ neurons derived from hLT-NES confirm proper mesDA neuron patterning on day 8. TUJ1 is included for visualization of neuronal projections. Scale bar, 100 μm. **c**, Validation of BMAL1 and EGFP overexpression in hLT-NES cells. Transgene expression was induced by doxycycline (2 µg/ml) for 24 h. Scale bar, 100 µm. **d**, Representative immunofluorescence images of LMX1A⁺Ki67⁺ cells derived from CTRL and BMAL1-OE hLT-NES cells confirm the presence of proliferating progenitors at day 6. Scale bar, 100 µm. **e**, EdU⁺γH2AX^−^ cells at day 6 indicate that EdU incorporation reflects progenitor proliferation rather than DNA damage. Cells treated with ionizing radiation (IR) are shown as a positive control for γH2AX staining. Scale bar, 50 μm. **f**, Western blot analysis of BMAL1 KD showing reduced BMAL1 protein levels in shBMAL1 compared to shCTRL hLT-NES. Lysates used for Western blot were collected at P2 after shRNA treatment. β-Actin was used as loading control. Uncropped Western blots provided as Source data. **g**, qPCR analysis of BMAL1 KD showing significantly reduced BMAL1 transcript levels in shBMAL1 versus shCTRL hLT-NES (p = 0.0087). Data are presented as relative fold change (ddCt) normalized to shCTRL from n = 3 independent differentiations. Individual data points, mean, and error bars representing s.e.m. are shown. ***p < 0.001, determined using two-sided Student’s t-test. **h**, Analysis of neurogenesis in shBMAL1 versus shCTRL hLT-NES on day 8 following continuous EdU labeling from day 4. Representative immunofluorescence images show cells undergoing neurogenesis (TH^+^EdU^+^, arrows). Scale bar, 100 µm. **i**, Box plots showing the percentage of TH^+^ neurons(shCTRL: 3.31 ± 0.52%; shBMAL1: 3.34 ± 0.23%, *p* = 0.97), cells undergoing neurogenesis (TH^+^EdU^+^/DAPI^+^; shCTRL: 0.31 ± 0.08%; shBMAL1: 0.07 ± 0.04%, *p* = 0.049), and proliferating cells (EdU^+^TH^−^/DAPI^+^; shCTRL: 38.8 ± 2.10%; shBMAL1: 27.7 ± 2.02%; mean ± s.e.m., *p* = 0.019) on day 8 following continuous EdU treatment from day 4 in shCTRL and shBMAL1 hLT-NES. **j**-**l**, Transgene expression was measured following BMAL1-OE on day 2 (**j**), day 4 (**k**) or day 6 (**l**). Arrows indicate Dox treatment timing. **m**, BMAL1 expression was measured following stable BMAL1-KD. Expression levels are shown as relative FC (ΔΔCt), normalized to day 0 ± s.e.m. from n = 3 independent differentiations. **n**, Gene expression was measured following BMAL1-OE or KD in WA09 on day 16. Expression labels are shown as relative FC (ΔΔCt), normalized to control. Box plots display interquartile range (IQR), median (indicated by line), and whiskers (1.5× IQR) with individual data points shown as a scatterplot. Asterisks indicate statistical significance (*p < 0.05; **p < 0.01; ***p < 0.001), determined using two-tailed Student’s t-test from n = 3 independent differentiations, 2 wells per condition. Source data

Our identification of *BMAL1* as a central TF in the Rgl1 neurogenic network led us to investigate its role in mesDA neurogenesis. While *BMAL1* is known to regulate adult neurogenesis, control cell cycle exit and circadian rhythms^46,47^, its function in the VM and mesDA neuron development is unknown. We first confirmed Bmal1 expression in the embryonic mouse VM, identifying Sox2^+^ cells coexpressing Bmal1 in the mFP at E13.5 (Extended Data Fig. 8a). To assess *BMAL1* function in human mesDA neurogenesis, we used human neuroepithelial stem (hLT-NES) cells (Sai2), which generate correctly patterned mesDA neurons by day 8 of differentiation (Extended Data Fig. 8b).

**Fig. 5:**
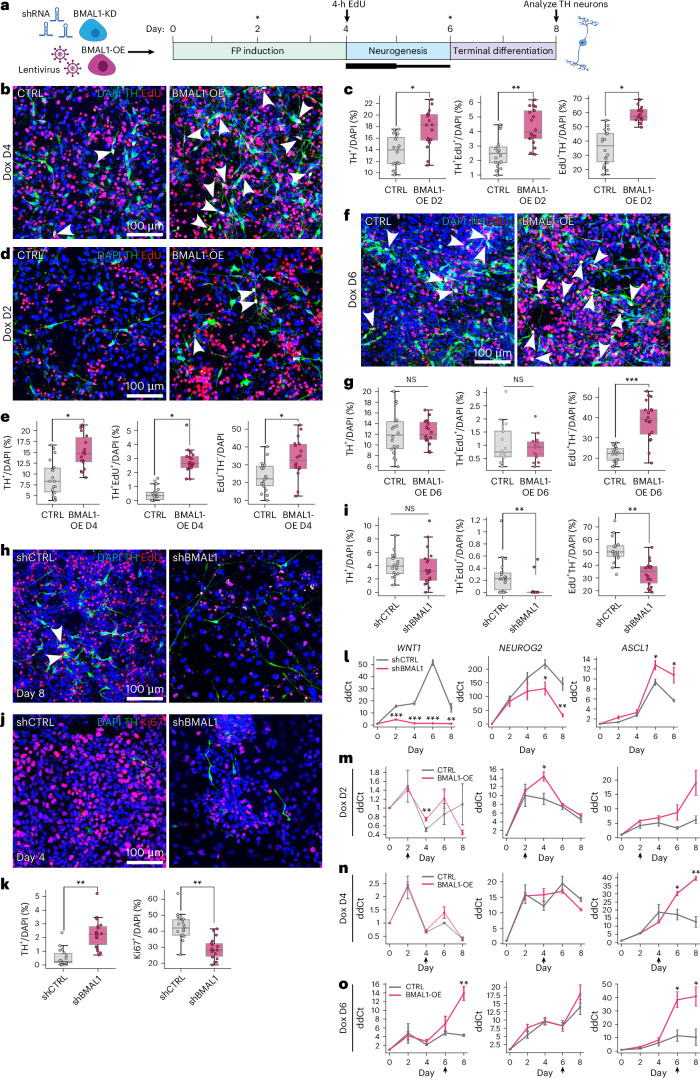
*BMAL1* regulates dopaminergic neurogenesis by modulating progenitor proliferation and differentiation in human neuroepithelial stem cells. **a**, Schematic of the differentiation protocol used to assess the role of BMAL1 in mesDA neurogenesis. BMAL1-KD and BMAL1-OE were performed in hLT-NES cells. BMAL1-OE was induced with doxycycline (Dox) at 2 μg ml^−1^ on day 4 and 1 μg ml^−1^ on day 5. Asterisks indicate additional BMAL1 induction days used in follow-up experiments. **b**–**g**, BMAL1-OE increases proliferation, neurogenesis and the yield of TH^+^ neurons. Representative immunofluorescence images showing Th^+^EdU^+^ neurons (arrowheads) in CTRL and BMAL1-OE hLT-NES. Cells were pulsed with 10 µM EdU for 4 h on day 2 (**d**), day 4 (**b**) or day 6 (**f**), followed by analysis on day 8. Arrowheads indicate cells undergoing neurogenesis (Th^+^EdU^+^) neurons. Box plots (**c**,**e**,**g**) showing the percentage of TH^+^ neurons^+^ (TH ^+^/DAPI^+^), neurogenic cells (TH^+^EdU^+^/DAPI^+^) and proliferating progenitors (EdU^+^TH^−^/DAPI^+^) in response to BMAL1-OE on day 4 (**c**), day 2 (**e**) or day 6 (**g**) in CTRL and BMAL1-OE hLT-NES, *n* = 3 independent differentiations, 2 wells per condition. TH⁺/DAPI⁺ = 17.72 ± 0.33% (BMAL1-OE) versus 13.67 ± 1.39% (CTRL), *P* = 0.047; TH⁺EdU⁺/DAPI⁺ = 4.30 ± 0.26% (BMAL1-OE) versus 2.52 ± 0.30% (CTRL), *P* = 0.020; EdU⁺/DAPI⁺ = 58.60 ± 1.66% (BMAL1-OE) versus 35.45 ± 6.79% (CTRL), *P* = 0.037; mean ± s.e.m. (**c**). TH⁺/DAPI⁺ = 15.19 ± 0.84% (BMAL1-OE) versus 9.11 ± 1.84% (CTRL), *P* = 0.040; TH⁺EdU⁺/DAPI⁺ = 2.78 ± 0.12% (BMAL1-OE) versus 0.47 ± 0.15% (CTRL), *P* = 0.0003; EdU⁺TH^−^/DAPI⁺ = 33.61 ± 3.20% (BMAL1-OE) versus 23.26 ± 1.54% (CTRL), *P* = 0.043; mean ± s.e.m (**e**). TH⁺/DAPI⁺ = 12.40 ± 0.36% (BMAL1-OE) versus 12.08 ± 2.12% (CTRL), *P* = 0.891; TH⁺EdU⁺/DAPI⁺ = 0.88 ± 0.14% (BMAL1-OE) versus 1.04 ± 0.44% (CTRL), *P* = 0.746; EdU⁺TH^−^/DAPI⁺ = 38.51 ± 1.44% (BMAL1-OE) versus 21.89 ± 0.36% (CTRL), *P* = 0.0004; mean ± s.e.m (**g**). NS, not significant. **h**–**k**, BMAL1-KD impairs DA neurogenesis and reduces progenitor proliferation. Representative immunofluorescence images of TH^+^EdU^+^ neurons in shCTRL and shBMAL1 hLT-NES following a 4-h EdU pulse on day 4, analyzed on day 8 (**h**). Arrowheads indicate cells undergoing neurogenesis (TH^+^EdU^+^) neurons. Box plots showing the percentage of TH⁺ neurons, neurogenic (TH⁺EdU⁺/DAPI⁺) and proliferating cells (EdU⁺TH^−^/DAPI⁺) on day 8 (**i**). EdU⁺TH^−^/DAPI⁺ = 33.55 ± 1.19% (shBMAL1) versus 51.46 ± 4.28% (shCTRL), *P* = 0.016; TH⁺/DAPI⁺ = 3.73 ± 0.46% (shBMAL1) versus 4.12 ± 0.62% (shCTRL), *P* = 0.638; TH⁺EdU⁺/DAPI⁺ = 0.05 ± 0.03% (shBMAL1) versus 0.27 ± 0.05% (shCTRL), *P* = 0.020; mean ± s.e.m. Representative immunofluorescence images of TH^+^ and Ki67^+^ cells in shCTRL and shBMAL1 hLT-NES on day 4. Box plots showing the percentage of TH⁺ neurons and proliferating cells (Ki67⁺/DAPI⁺) on day 4 (**k**). Ki67⁺/DAPI⁺ = 28.91 ± 2.31% (shBMAL1) versus 42.52 ± 2.43% (shCTRL), *P* = 0.015; TH⁺/DAPI⁺ = 2.28 ± 0.39% (shBMAL1) versus 0.54 ± 0.11% (shCTRL), *P* = 0.013; mean ± s.e.m. Box plots display IQR, median (line), whiskers (1.5× IQR) and individual data points (scatter) (**h**,**k**). Asterisks indicate statistical significance (*P < 0.05; **P < 0.01; ***P < 0.001), determined using two-tailed Student’s *t* test. *n* = 3 independent differentiations, two wells per condition. **l**–**o**, qPCR analysis following BMAL1-OE or BMAL1-KD. Gene expression was measured after BMAL1-OE on day 2 (**m**), day 4 (**n**), day 6 (**o**) or stable BMAL1-KD (**l**). Expression levels are shown as relative FC (ΔΔCt), normalized to day 0 ± s.e.m. from *n* = 3 independent differentiations. Arrows in **m**–**o** indicate Dox treatment timing. Asterisks denote statistical significance (*P < 0.05; **P < 0.01; ***P < 0.001), determined using two-tailed Student’s *t* test, with exact *P* values provided in Supplementary Table 2. FC, fold change. Source data

We generated stable doxycycline-inducible *BMAL1* or *EGFP* hLT-NES lines (Extended Data Fig. 8c), which induced robust *BMAL1* expression during peak mesDA neurogenesis (2 µg ml^−1^ and 1 µg ml^−1^ doxycycline on days 4 and 5, respectively). The experimental setup is summarized in Fig. 5a. A 4-h EdU pulse at day 4 revealed that *BMAL1* induction increased both the proportion of proliferating progenitor cells (EdU^+^/DAPI^+^; +65%) and cells undergoing mesDA neurogenesis (EdU^+^TH^+^/DAPI^+^; +72%), resulting in a 22% increase in TH^+^ neuron yield by day 8 (Fig. 5b,c). To examine potential temporal effects, we induced *BMAL1* before (day 2) and after (day 6) peak neurogenesis. Although *BMAL1* significantly increased progenitor proliferation at both time points (by 44% and 76%, respectively), only early *BMAL1* induction significantly increased mesDA neurogenesis (491%) and TH^+^ neuron yield (67%; Fig. 5d–g). To validate that EdU incorporation after peak neurogenesis represented true progenitor proliferation rather than DNA damage, we confirmed the presence of LMX1A⁺Ki67⁺ progenitors and the absence of γH2AX^+^ nuclei at day 6 (Extended Data Fig. 8d,e).

To assess *BMAL1* loss of function, we generated stable *BMAL1* knockdown (KD) hLT-NES cells using shRNA (shBMAL1) and confirmed reduced BMAL1 protein and RNA levels relative to a control shRNA line (shCTRL; Extended Data Fig. 8f,g). EdU labeling on day 4 showed that *BMAL1* KD reduced progenitor proliferation by 35% and mesDA neurogenesis by 80%. However, the overall proportion of TH^+^ neurons on day 8 was unchanged (Fig. 5h,i). To determine whether this decrease resulted from premature cell cycle exit and early mesDA neurogenesis, we first ruled out label dilution with continuous EdU labeling between days 4 and 8 (Extended Data Fig. 8h,i). We then analyzed day 4 cells before the EdU pulse and observed fewer Ki67^+^ progenitor cells (−32%) and a 322% increase in TH^+^ neurons, indicating that a loss of *BMAL1* leads to early cell cycle exit, consistent with reports in other cell types^48,49^, resulting in earlier mesDA neurogenesis.

### *BMAL1* modulates Wnt signaling to regulate the timing of mesDA neurogenesis

To investigate how *BMAL1* regulates the balance between proliferation and neurogenesis during mesDA differentiation, we examined its influence on genes that control progenitor fate. *BMAL1* has previously been shown to regulate Wnt signaling^50,51^, a pathway that has a critical role in mesDA progenitor proliferation and neurogenesis^52^. Consistent with this, the mRgl1 transcriptional network revealed putative interactions between *BMAL1* and Wnt pathway components.

We first validated the efficiency of *BMAL1* overexpression (OE) and KD throughout the differentiation (Extended Data Fig. 8j–m). qPCR analysis of *WNT1* expression revealed that *BMAL1* OE significantly increased *WNT1* levels, while *BMAL1* KD decreased them, supporting a role for *BMAL1* in regulating Wnt signaling during mesDA differentiation (Fig. 5l–o). We then examined the expression of *NEUROG2* and *ASCL1*, two proneurogenic TFs known to control mesDA neurogenesis^34^, and regulated by Wnt signaling^53^. Early *BMAL1* induction (day 2) upregulated *NEUROG2* expression, while later *BMAL1* induction (days 4 and 6) preferentially increased *ASCL1* levels, suggesting that *BMAL1* modulates neurogenic TF expression in a temporally dynamic manner (Fig. 5m–o). Conversely, *BMAL1* KD reduced *NEUROG2* expression from day 6 onward but increased *ASCL1* levels during the same period (Fig. 5l), potentially reflecting context-dependent *BMAL1* function and/or compensatory *ASCL1* upregulation^34^. To validate these findings, we analyzed *WNT1*, *NEUROG2* and *ASCL1* expressions during mesDA neuron differentiation of hESCs and observed similar BMAL1-dependent modulation on day 16 (Extended Data Fig. 8n).

Together, these results identify *BMAL1* as a new regulator of mesDA neurogenesis, likely acting with Wnt signaling to modulate the timing of neurogenic TF expression.

### Single-cell lineage tracing reveals hRgl1 as the shared progenitor of human mesDA neuroblasts and hRgl3

**Fig. 6:**
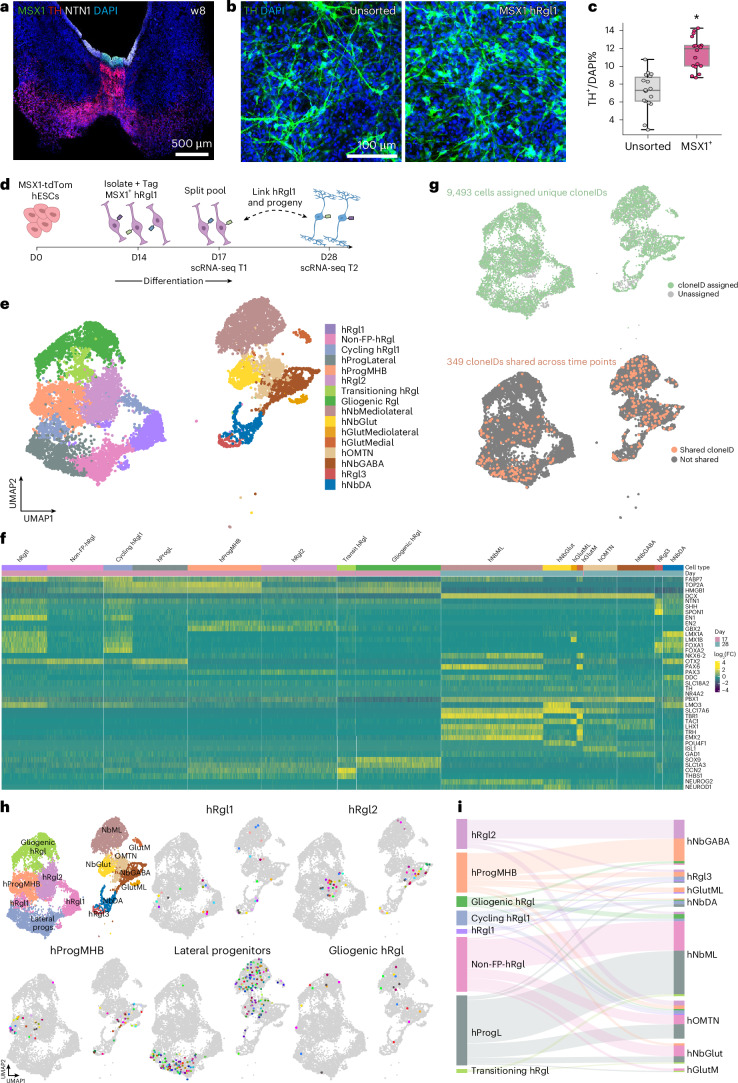
Single-cell lineage tracing reveals hRgl1 as the progenitor of mesDA neurons. **a**, Representative immunostaining showing the expression of *MSX1* (hRgl1), *NTN1* (hRgl3) and *TH* (mesDA neurons) in the developing human VM at embryonic week 8, detected with RNAScope. **b**, Representative immunofluorescence images of TH^+^ neurons on day 28 of differentiation, following FACS enrichment for MSX1-tdTomato⁺ cells at day 14. **c**, Box plots showing the percentage of TH⁺ neurons after enrichment for the hRgl1 marker MSX1-tdTomato on day 1 (right) versus unsorted control. TH⁺/DAPI⁺ = 11.46 ± 0.86% (tdTom⁺) versus 7.21 ± 1.14% (unsorted), *P* = 0.041; mean ± s.e.m. Box plots display IQR, median (line), whiskers (1.5× IQR) and individual data points (scatter). Asterisks indicate statistical significance (*P < 0.05), determined using two-tailed Student’s *t* test from *n* = 3 independent differentiations. **d**, Schematic overview of the single-cell lineage tracing workflow used to trace hRgl1 progeny. **e**, UMAP showing cell types identified at days 17 and 28 of differentiation, following MSX1-tdTomato^+^ enrichment on day 14. **f**, Heatmap displaying the expression of selected regional and functional marker genes used to support cell-type annotation. **g**, UMAP showing cells with identified cloneIDs at days 17 and 28, and multicellular (>2) cloneIDs shared across time points. **h**, UMAPs showing projected lineage relationships among progenitor populations on day 18 and their progeny at day 28. Progenitor populations were summarized for simplicity based on anatomical location and/or function. hFP-Rgl1, cycling and hFP-Rgl1; lateral progenitors, non-FP-hRgl and hProgL; gliogenic progenitors, transitioning and gliogenic hRgl. Cells are colored by cloneID, with each color representing a unique multicellular clone shared across both time points. **i**, Sankey plot summarizing projected lineage relationships among progenitor populations at day 17 and their differentiated progeny at day 28. Source data

**Extended Data Fig. 9:**
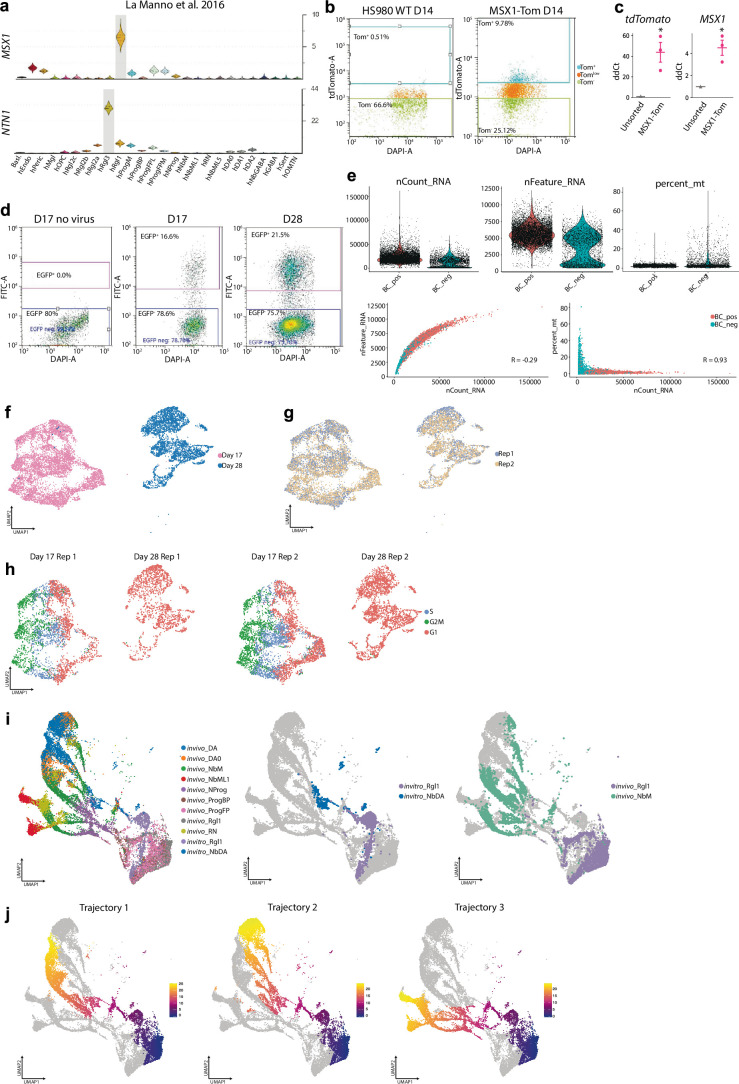
Single-cell lineage tracing of hRgl1. **a**, Violin plots showing expression of *MSX1* (hRgl1) and *NTN1* (hRgl3) across all cell types in the developing human VM (ref. ^6^). **b**, Fluorescence-activated cell sorting (FACS) of WT HS980 hESCs (left) and MSX1-tdTomato HS980 hESCs (right) on day 14 of differentiation. Cells were classified as tdTom^−^, tdTom^low^, or tdTom^+^ based on fluorescence intensity, with the tdTom^+^ fraction collected. The fraction of tdTom⁺ cells in MSX1-tdTomato HS980 from three independent differentiations was 7.98 ± 1.69% (mean ± s.d.). **c**, qPCR validation of *tdTomato* and *MSX1* enrichment following FACS sorting on day 14. Expression levels are shown as relative fold change (ΔΔCt), normalized to day 0 and presented as mean ± s.e.m. from *n* = 3 independent differentiations (*tdTomato* p = 0.047; *MSX1* p = 0.036, determined by two-tailed Student’s t-test). **d**, FACS plots showing percentage of EGFP^+^ cells in a non-transduced control (left) and MSX1-tdTomato⁺ cells transduced with lentivirus on day 14, analyzed at day 17 (middle) and day 28 (right) of differentiation. Color reflects cell density, increasing from black (lowest) to red (highest). Fraction of EGFP⁺ cells at day 17 was 14.31 ± 0.45%, and at day 28 was 22.01 ± 1.07% (mean ± s.d.; n = 2 independent differentiations). **e**, Quality control metrics for single-cell RNA-seq data: nCount_RNA, nFeature_RNA, and percent mitochondrial reads (percent_mt), stratified by barcode (BC) status. Bottom: correlations between metrics. **f**, UMAP showing all cells colored by differentiation day. **g**, UMAP showing all cells colored by biological replicate. **h**, UMAPs showing cell cycle phase distribution across replicates and time points. **i**, UMAP of in vitro hRgl1 and hNbDA integrated with developing in vivo midbrain data from ref. ^4^. Middle: in vitro hRgl1 and hNbDA highlighted. Right: endogenous hRgl1 and hNbM highlighted. **j**, Slingshot pseudotime analysis showing three major trajectories from hRgl1: trajectories 1 and 2 progress through hNbDA to mesDA neurons, while trajectory 3 leads to mediolateral midbrain populations.

To investigate the lineage relationship between hRgl1 and mesDA neurons, we used a fluorescent hESC-reporter line (MSX1-tdTomato) to enrich hRgl1 cells based on *MSX1* expression^54^. RNAScope analysis of human fetal VM tissue at 8 weeks postconception confirmed *MSX1* expression in the mFP and revealed the spatial relationship between hRgl1 (*MSX1*^+^), hRgl3 (*NTN1*^*+*^) and *TH*^+^ mesDA neurons (Fig. 6a and Extended Data Fig. 9a).

Differentiation of MSX1-tdTomato^+^ hESCs followed by fluorescence-activated cell sorting (FACS) isolation of MSX1^+^ cells on day 14 (Extended Data Fig. 9b,c) resulted in a 60% increase in TH^+^ neuron yield by day 28 compared to an unsorted control (Fig. 6b,c). To directly trace the lineage of hRgl1, we then isolated MSX1-tdTomato^+^ cells on day 14 and labeled them with a lentiviral library encoding unique 30-bp cloneIDs (EF1a-H2B-EGFP-cloneID)^55^. Barcoded EGFP⁺ cells were isolated on days 17 and 28 from two independent differentiations (Extended Data Fig. 9c,d) and analyzed using scRNA-seq (Fig. 6d).

After quality control (Extended Data Fig. 9e–h), we obtained 8,362 and 4,623 high-quality cells from days 17 and 28, respectively, that were annotated based on scRNA-seq data from the developing human VM^4^ and expression of regional and functional marker genes. At day 17, we identified cells with strong transcriptional similarity to hRgl1 and hRgl2, as well as progenitors at the midbrain–hindbrain boundary (hProgMHB), dorsal progenitors (non-FP-hRgl and hProgL) and transitioning or gliogenic Rgl (Fig. 6e,f). By day 28, the cultures were enriched for midbrain neuroblasts and neuronal populations, including dopaminergic, glutamatergic and GABAergic neuroblasts, as well as glutamatergic neurons and neurons of the oculomotor and trochlear nuclei (OMTN; Fig. 6e,f).

To reveal lineage relationships among cell types, we recovered 6,902 and 3,705 barcoded cells on days 17 and 28, respectively, corresponding to 349 unique multicellular cloneIDs shared across time points (Fig. 6g). These cross-time-point cloneIDs revealed clonal relationships among cell types, identifying hRgl1 as the progenitor of mesDA neuroblasts and OMTN neurons, two neuronal populations arising from the ventral-most aspect of the mFP (Fig. 6h,i). Consistent with this, integration with endogenous midbrain data^4^ followed by pseudotime analysis revealed three major trajectories from hRgl1—two progressing through hNbDA and culminating in mesDA neurons, and the third giving rise to neuronal populations arising lateral to the mesDA neurons in the mFP (Extended Data Fig. 9i,j).

We also observed clonal relationships between hProgMHB and mesDA neuroblasts, while dorsal progenitors, including hRgl2, non-FP hRgl and hProgL, gave rise to lateral midbrain neuronal populations, such as the mediolateral neuroblast (NbML), NbGABA, glutamatergic and OMTN neurons. Notably, hRgl3 cells only emerged on day 28 in our cultures and were predominantly derived from hRgl1 and hProgMHB, identifying hRgl1 as the shared progenitor of mesDA neuroblasts and the niche cell that supports their development.

Together, these findings reveal lineage relationships between spatially distinct progenitor populations and their neuronal progeny in the developing VM, and establish hRgl1 as the progenitor of both the mesDA lineage and their supporting niche cell, hRgl3.

## Discussion

Rgl are a heterogeneous population with diverse roles in central nervous system development, but the extent of their functional specialization remains poorly understood. Here we integrate bulk and scRNA-seq data from the developing mouse VM and identify two subtypes of mFP Rgl (mRgl1 and mRgl3) with specialized roles in mesDA neuron development that are largely conserved across species. Analyzing their individual transcriptional profiles^4^ revealed distinct functional signatures, suggesting a role for mRgl1 in neurogenesis and mRgl3 in signaling and ECM organization. Moreover, subtype-specific TF networks identified distinct gene regulatory programs that further supported their functional specialization. Finally, single-cell lineage tracing during human mesDA differentiation revealed hRgl1 as the bona fide progenitor of both mesDA neuroblasts and hRgl3.

Our analysis of mRgl3 identified a TF network associated with ventral midline formation and dorsoventral patterning^56^, suggesting a signaling role for mRgl3 at the ventral midline. Accordingly, CellChat analysis predicted Rgl3 to have the highest number of outgoing signaling interactions in both species, expressing ligands predicted to target all cell types of the mesDA lineage. To functionally assess the role of hRgl3-expressed factors on mesDA neuron development in vitro, selected factors were added to differentiating cells during the stage when hRgl3 emerges in the culture^26^. However, although these factors were selected based on transcriptomic data demonstrating their expression in hRgl3, they were not directly derived from hRgl3 cells and may therefore not fully recapitulate endogenous hRgl3 signaling. Nonetheless, while not all ligands improved differentiation outcomes, we identified new factors that enhanced TH⁺ neuron yield and cell survival in vitro, and found that inhibition of receptors for hRgl3-expressed ligands reduced TH⁺ neuron numbers, supporting a functional role for these signaling pathways during mesDA neuron development. Notably, hRgl3-expressed ligands of the renin-angiotensin pathway, and blocking the angiotensin receptor AGTR1 reduced survival and TH^+^ neuron numbers, suggesting a role for hRgl3-derived *AGT* in embryonic mesDA neuron survival. This is intriguing as *AGTR1+* mesDA neurons in the human SNc are particularly vulnerable in PD^27^, the angiotensin pathway is implicated in both PD pathology^57^ and DA neurotransmission^58^, and AngII protects rat mesDA neurons from rotenone-induced cell death^59^. Similarly, we demonstrate that hRgl3-expressed ECM proteins NTN1 and SLIT1 enhance survival and increase TH^+^ neuron yield in vitro. While *Ntn1*, *Slit1* and their corresponding receptors are expressed in the developing rodent midbrain^20,21^ and Ntn1 has been shown to support mesDA neuron survival in the adult mouse SNc^60^, our results provide evidence of the survival-promoting effects of NTN1 and SLIT1 in human embryonic development and highlight the ECM as a valuable source of developmental factors that could be tailored to specific cell types to improve differentiation outcomes. Collectively, our results identify Rgl3 as a signaling center in the developing mFP of both species, expressing both previously characterized and new factors that improve the yield and survival of TH⁺ neurons in vitro.

Conversely, Rgl1 was identified as a neurogenic cell type in both species, defined by a transcriptional state driven by proneurogenic TFs. Notably, we identified *BMAL1* as a central node in the mRgl1 neurogenic network, and functional validation revealed this TF as a new regulator of human mesDA neurogenesis, influencing the balance between proliferation and neurogenesis through the Wnt signaling pathway. Interestingly, *BMAL1* KD was recently shown to induce mesDA neurodegeneration^61^, and its expression is reduced in peripheral blood mononuclear cells of PD patients^62^, suggesting that *BMAL1* contributes to both mesDA neuron development and survival. Finally, we traced the progeny of hRgl1 in vitro and identified hRgl1 as the shared progenitor of mesDA^13^ neuroblasts and hRgl3, demonstrating that hRgl1 gives rise both to the mesDA lineage and the niche cell type that supports its development.

Together, our in vivo and in vitro analyses reveal distinct, specialized functions for mFP Rgl subtypes in mesDA neuron development. We identify Rgl1 as the shared progenitor of the mesDA lineage and hRgl3, and show that enriching for hRgl1 increases TH⁺ neuron yield, while ligands secreted by Rgl3 enhance mesDA neuroblast survival. These findings establish a lineage relationship between mesDA neuroblasts and their supporting niche, identify Rgl1 and Rgl3 as complementary regulators of mesDA neuron development, and offer a promising strategy for improving cell quality in regenerative approaches for PD.

## Methods

### Bulk RNA sequencing

Mouse embryos were obtained from TH-GFP animals^63^ that were mated overnight, and the plug was considered E0.5 at noon the following day. Embryos were dissected out of the uterine horns at E11.5–E14.5 and placed in ice-cold sterile PBS, where brain regions were dissected under a stereomicroscope with an ultraviolet attachment to detect GFP. Tissue samples were collected in separate tubes and stored at −80°C until RNA isolation. Ethical approval for mouse experimentation was granted by the local ethics committee, Stockholm Norra Djurförsöksetiska Nämnd N326/12 and N158/15.

Total RNA was extracted using the RNeasy kit (Qiagen), and RNA concentration and integrity were assessed using Qubit and 2200 TapeStation (Agilent). Libraries were prepared following Illumina TruSeq protocols and sequenced on an Illumina HiSeq 2000, generating single-end 50-bp reads. Sequencing depth ranged from ~1.2 million to ~28.4 million reads per sample, with an average depth of ~9.2 million reads. Each developmental stage (E11.5–E14.5) was sequenced with *n* = 3 biological replicates per condition. Sequencing reads were demultiplexed and mapped to the UCSC mm10 mouse genome using Bowtie. Gene expression was normalized and reported as reads per kilobase of transcript per million mapped reads (RPKM). RNA-seq data are publicly available in the NCBI GEO database under accessions GSE82099 (E12.5) and GSE117394 (E11.5, E13.5 and E14.5).

### Bulk gene expression analysis

DEGs were identified using Qlucore Omics Explorer (v3.1; Qlucore AB), comparing VM samples to other brain regions. A *t* test with false discovery rate (FDR) correction was applied, using a variance filtering threshold of 15% and a significance threshold of *P* = 0.01 unless otherwise stated. Sample correlation was calculated from log_2_(RPKM + 1) transformed expression values, with an additional variance filtering of 12.5% applied before analysis. Significant expression was defined as exceeding a baseline of 99.8% posterior probability. All statistical analyses were conducted in the R Package (v3.4.0). The dplyr (v0.5.0) and reshape2 (v1.4.2) were used for data filtering and manipulation, PCA was performed using FactoMineR (v1.35), hierarchical clustering with vegan (v2.4-2) and GO term analysis with ClusterProfiler (v3.4.5). Data visualization was performed using ggplot2 (v2.2.1), and heatmaps were generated with the pheatmap package (v1.0.8).

### WGCNA

WGCNA^64^ was performed on log_2_(RPKM + 1) transformed values, filtered by variance until 10,068 genes were selected. The topological overlap matrix was calculated with the variables of a signed network, with power of 7, as this is the lowest power that results in a scale-free network^65^. The identification of modules was performed with the ‘tree’ option on a minimum module size of 100; modules with correlation higher than 90% were merged. Module enrichment for DEG in VM across all analyzed stages was performed using Fisher’s exact test with FDR *q*-value correction^66^. A module enrichment score (ES) was calculated as the product between the −log_10_(*q*) and a standardized *z* score for DEGs per module. Hub genes were identified based on module membership scores and gene significance values. Module network layout was made using Cytoscape and interactions in the top 5% correlation (from WGCNA) were selected for further analysis. Node colors represent expression changes over time, calculated as the normalized RPKM difference between late (E13.5, E14.5) and early (E11.5, E12.5) stages.

VM gene modules with developmental or stage-dependent expression were identified using WGCNA on VM samples from E11.5 to E14.5. Further filtering for genes not expressed in the VM was performed until 9,061 genes were selected. The topological overlap matrix was calculated with the options of a signed network, with power of 17. Modules were defined using the tree-clustering method (minimum module size = 100 genes) and merged when correlation exceeded 99%. Correlation with the samples’ trait and Student asymptotic *P* values were calculated as described in ref. ^64^. Embryonic stage (E11.5 to E14.5) was used as sample trait, ordinal values for stage (1 for E11.5, 2 for E12.5 and so on) and binary values for middle stages (E12.5, E13.5 as 1). Network layouts and analysis were performed using Cytoscape (v3.3.0)^67^ or Gephi (v0.9.1)^68^. WGCNA was performed in R with the WGCNA package (v1.49)^64^.

### Network single-cell deconvolution

Using data from ref. ^6^, genes in the network were assigned to cell types based on expression above baseline threshold (see above). Genes were assigned to cell types based on binary presence/absence in single-cell profiles. Cell types represented by a single gene or contributing to less than 1% of the total assigned genes were excluded from the network.

### Single-cell GSEA

GSEA of mouse cell types was performed by preranking genes based on the class difference for the cell type of interest. The analysis was performed on the GSEA software (v2.2.2)^69^ with the MSigDB gene sets (v5.0) for canonical pathways and GO biological processes^69^ with 1,000 permutations. Due to the nature of the single-cell transcriptome profiles and molecular counting with unique molecular identifiers,^70^ negative ESs on a gene set for a cell type in particular were interpreted as enrichment in another compared cell type. Analysis of GO enrichment was performed using the R package ClusterProfiler^71^ or MSigDB^69^ with FDR *q*-value correction. Enrichment of the targets of TF were analyzed with hypergeometric test and with FDR *q*-value correction^66^ over the MSigDB C2 gene sets.

GSEA of human Rgl cell types—mRgl1–mRgl3 gene sets were defined by selecting all genes expressed above baseline (>99.8% posterior probability) in mRgl1–mRgl3 and then selecting only those genes uniquely expressed by each cell type. Genes related to sex, cell cycle, blood, mitochondria and immediate early genes were removed from human Rgl transcriptomes before the analysis. Analysis was performed using GSEApy^72,73^ with prerank module with 1,000 permutations. The prerank input was obtained from performing DEG among human Rgl cell types using scanpy’s rank_genes_groups with method = ‘Wilcoxon’ on log-normalized expression matrix.

### Single-cell TF pattern mining

TFs expressed above threshold^6^ in each Rgl cell type were used as input to obtain the target of the human homologous TF from the iRegulon (v1.3) plugin for Cytoscape^74^, retrieving up to 1,000 target genes per TF. TFs were clustered based on shared target genes using the Jaccard index as a measure of similarity.

Enrichment analysis of combinatorial TF target genes was performed using the FWY permutation procedure (v1.0.1) with 1,000 permutations^29,75^ and a significance threshold of 0.05 in the one-tailed Fisher test. TF target gene expression among Rgl cell types was scored using the logarithmic difference among the groups^69^, with values above 0.5 (or 50% of upregulation) considered enriched in that cell type. The resulting significant combinatorial patterns were represented as a network on which the edge between a set of TFs is measured by an interaction score. TF pairs were assigned an interaction score calculated as the sum of –log_10_(adjusted *P* value) across all shared TF combinations. Values of <0.001 were set to 0.001 for interaction score calculations.

A control null distribution was performed by selecting random TFs from the MSigDB C3 database^69^, selecting the same number of TFs used before for Rgl1–Rgl3, respectively. For each cell type, 100 random TF sets were analyzed using FWY, with all other parameters held constant.

### CellChat analysis

To investigate potential interactions among cell types in the mouse and human VM, cell-to-cell communication analysis was performed using CellChat^76^ (v1) on the datasets published in refs. ^4,6^. To enrich for neurodevelopmentally relevant ligand–receptor pairs, the CellChat ligand–receptor database was merged with the CellPhoneDB^77^ and CellTalkDB^78^ databases. This extended database assumes conservation of ligand–receptor pairs between mouse and human and simplifies some multisubunit and antagonist interactions modeled by CellChat, as these are not available for all pairs in the extended database. For the mouse dataset, we combined all time points into a single analysis, given the limited cell count, disregarding spatiotemporal proximity among cell types. The mouse dataset (excluding unannotated and endothelial cells) was analyzed following the authors’ workflow:

(identifyOverExpressedGenes, identifyOverExpressedInteractions, projectData, computeCommunProb, filterCommunication, subsetCommunication, netAnalysis_computeCentrality), with min.ncells = 10.

To avoid calling signaling activity by lowly expressed genes, gene expression was filtered as in ref. ^6^ to retain only those genes expressed above baseline for any given cell type independently in each cell type. The number of outgoing interactions was calculated by counting the ligand–receptor pairs for which a given cell type is a source and the number of incoming interactions by counting the ligand–receptor pairs for which a given cell type is a target. Before plotting chord plots, we manually removed the ligand–receptor pair from the following interactions: Wnt5a-Notch1, and all interactions involving App, Adam10, Adam17, Mmp9, Tyrobp, Ntng, as the support in the literature for these interactions is limited. The CellChat plotting functions were also modified to allow for coloring chord plot arrows by target cell type instead of source cell type. To plot the heatmaps, we aggregated ligand–receptor pairs into single families (Notch, Dlk, Fgf, Igf2, Nrxn, Slit, Sema) by summing communication probabilities.

For the human data, we analyzed cells with midbrain regional annotation (see ref. ^4^ for details) following the same steps as above, without filtering based on baseline gene expression. Instead, we prioritized top interactions by thresholding with a minimal communication probability of 3%. To isolate cell types with an FP annotation, cells were scored positively based on *LMX1A*/*FOXA2*/*EN1* expression and negatively based on *PITX2*/*NKX6-1*/*NKX2-2* expression, choosing hard thresholds consistent with the clusters in Uniform Manifold Approximation and Projection (UMAP) space. The 95th percentile of the bootstrapped distribution of the combined incoming and outgoing interaction scores was calculated by removing the source and target components for each signaling axis (represented as source | target | ligand | receptor) identified by CellChat and repopulating the source and target by selecting a cell type from the same tercile of ligand/receptor expression, using a random uniform distribution. This process was iteratively performed 10,000 times for all source | target | ligand | receptor rows, generating bootstrap replicas. Cell type occurrences as both source and target were tallied for each bootstrap replica, producing a compound score that reflects the overall centrality in signaling.

To support our cell–cell communication predictions, we applied LIANA^79^ through its Python API, using either the prebuilt LIANA ligand–receptor database or our merged reference database. Analyses were run with an expression proportion threshold of 0.05. Ligand–receptor pairs with specificity ranks of >0.2 or a magnitude rank of 1 were excluded. Downstream analyses were repeated in the same way as for the CellChat analysis.

The code and datasets are available on GitHub at https://github.com/lamanno-epfl/rgl3_signaling_dopaminergic_analysis/tree/main.

### ECM cell-type score

Cell-type contribution to ECM was calculated with scRNA-seq data from ref. ^6^. ECM score was calculated based on the expression of ECM core genes and genes contributing to ECM regulation as defined by the Matrisome project^17^.

The score for a gene set is calculated as:

$${S}_{\mathrm{gset},\mathrm{ct}}=\,\frac{{\sum }_{g\in \mathrm{gset}}{M}_{g,\mathrm{ct}}}{{\sum }_{c\in \mathrm{ctypes}}{\sum }_{g\in \mathrm{set}}{M}_{g,c}}+\,\frac{{\sum }_{g\in \mathrm{gset}}{N}_{g,\mathrm{ct}}}{{\sum }_{c\in \mathrm{ctypes}}{\sum }_{g\in \mathrm{set}}{N}_{g,c}}$$

where ${M}_{g,\mathrm{ct}}$ is the Bayesian estimate of the expression level of the gene *g* in cell type ct and ${N}_{g,\mathrm{ct}}$ is the value of the indicator vector that is 1 when the gene *g* is expressed in cell type ct above baseline and 0 otherwise. The score is an indicator of the diversity of genes in a biological function or gene set and the expression levels of those genes. The combined score, obtained by summing both components, gives us an ECM score. The threshold line was set at the 99th percentile of the bootstrapped distribution of the mean of the combined score with 10 × 10^5^ replicates.

### Analysis of human scRNA-seq data

Analysis of human developing VM was performed on the dataset published in ref. ^4^. For information on how cell-type identity was assigned based on the cell types as defined in ref. ^6^, see ref. ^4^. To analyze the neurogenic potential of FP Rgl1, we only considered the expression of neurogenic genes in Rgl1 with FP (*LMX1A*^*+*^*FOXA2*^*+*^*EN1*^*+*^) identity. For plotting, the log expression of the product of any combination of three or more neurogenic genes (*BMAL1*, *E2F3*, *E2F5*, *ASCL1*, *NEUROG2* and *SOX5*), five BMAL1 downstream targets (*BMP7*, *DVL2*, *NEUROD1*, *GSK3B*, *TCF12*, *NR4A2*, *HDAC* and *IGF1R*) and all four postmitotic mDA neuron genes (*EN1*^*+*^, *NR4A2*^*+*^, *TH*^*+*^ and *PITX3*^*+*^) were plotted onto the UMAP.

### Human neuroepithelial stem cell differentiation

Sai2 hLT-NES cells were maintained as previously described^80^ for hLT-NES. For differentiation, hLT-NES cells were dissociated (TrypLE Select; Thermo Fisher Scientific, 12563011) and seeded at a density of 150,000 cells per cm^2^ on glass coverslips coated with poly-L-ornithine (0.02 mg ml^−1^; Sigma-Aldrich, P4957) and laminin (2 μg cm^−2^; Invitrogen, 23017-015). Cells were cultivated in DMEM/F12 (Gibco, 31331028) with 10% FBS. On days 0–1, N2 (1:100; Gibco, 17502001), B27 (1:1,000; Gibco, 17504001), SHH (200 ng ml^−1^; R&D, 461-SH-025), CT99021 (1 μM; Sigma-Aldrich, SML1046) and WNT5A (100 ng ml^−1^; R&D, 645-WN-010) were added to the culture media. The concentration of B27 was increased (1:100) between days 2 and 3. On days 4–5, culture media were supplemented with N2 (1:100), B27 (1:100), SHH (50 ng ml^−1^), GDNF (20 ng ml^−1^; R&D, 212-GD-050) and WNT5A (100 ng ml^−1^; R&D, 645-WN-010). On days 6–7, B27 and SHH were removed from the culture media and BDNF (20 ng ml^−1^; R&D, 248-BDB-050), ascorbic acid (200 µM; Sigma-Aldrich, A4403), TGFβ3 (1 ng ml^−1^; R&D, 243-B3-010) and dbcAMP (0.5 mM; Sigma-Aldrich, D0627) were added.

### BMAL1-KD and BMAL1-OE

For BMAL1-KD, commercially available BMAL1 shRNA lentiviral particles targeted against the human transcript were used (Santa Cruz Biotechnology, sc-38166-V) to generate stable hLT-NES cell lines selected with puromycin (500 ng ml^−1^ first 4 days, maintained with 200 ng ml^−1^). A nontargeting shRNA control (Santa Cruz Biotechnology, sc-108080) was used as a negative control. BMAL1-KD was confirmed by qPCR and western blot using anti-BMAL1 (1:1,000; ab93806) and anti-β-ACTIN (1:5,000; ab6276) as a loading control. Differentiation experiments were performed using stable hLT-NES shRNA cells within three passages of lentiviral infection. Cells were pulsed with 10 µM EdU (Invitrogen, C10337) for 4 h on day 4, or continuously from days 4 to 8.

For BMAL1-OE, the coding sequence of mouse Bmal1 was synthesized (Twist Biosciences) and cloned into the NheI site of FUW–TetO–MCS (Addgene, 84008; kindly donated from S. Piccolo). The final plasmid was verified by sequencing before usage. The TetO–FUW–EGFP was obtained from Addgene (30130). Lentiviral production was performed as previously described^81^. BMAL1-OE was verified by immunocytochemistry using anti-BMAL1 (1:1,000; ab93806). BMAL1 expression was induced during hLT-NES differentiation using doxycycline (2 µg ml^−1^ on the first day of induction and 1 µg ml^−1^ the following day, starting on either day 2, 4 or 6; Sigma-Aldrich). Cells were pulsed with 10 µM EdU (Invitrogen, C10337) for 4 h, coinciding with the first day of doxycycline treatment.

To assess potential DNA damage, hLT-NES cells were exposed to ionizing radiation to validate the specificity of the γH2AX antibody. Irradiation was performed using a CIX2 Xstrahl X-ray machine (Xstrahl) at 195 kV and 10 mA with a 3-mm aluminum filter and a 40-cm focus-to-specimen distance. Cells received a total dose of 10 Gy, delivered at ~1.3 Gy min^−1^.

BMAL1-KD and BMAL1-OE hESC experiments were performed from day 11 of differentiation. Changes in BMAL1 expression were validated by qPCR. Between days 14 and 16, BMAL1-KD hESCs were treated with 100 ng ml^−1^ puromycin and BMAL1-OE hESCs were treated with 2 µg ml^−1^ doxycycline.

### hESC differentiation

WA09 hESCs were obtained from WiCell, maintained and differentiated as previously described^82^, with the addition of SHH-C24II (200 ng ml^−1^; Miltenyi Biotec, 130-095) between days 7 and 10 of the differentiation protocol. On day 22, cells were prepared for ligand and ECM testing. Cells were dissociated (TrypLE Select) and seeded at a density of 500,000 cells per cm^2^ in 96-well plates. For ligand testing, cells were cultured in maturation media(cit) supplemented with WNT5A (100 ng ml^−1^; R&D, 645-WN-010), IWP2 (10 µM; Tocris, 3533), Valsartan (10 µM; Sigma-Aldrich, SML0142), AngII (100 nM; Sigma-Aldrich, A9525), BMP1 (100 ng ml^−1^; R&D, 1927-ZN-010), FGF7 (100 ng ml^−1^; R&D, 251-KG), NBL1 (100 ng ml^−1^; R&D, 755-DA-050), DKK3 (100 ng ml^−1^; R&D, 1118-DK-050) or CGP-42112A (300 nM; Sigma-Aldrich, C160) between days 22 and 35. Media containing ligands was replaced every other day. For ECM experiments, plates were coated with SPARC (R&D, 941-SP-050), NTN1 (R&D, 6419-N1-025), SLIT1 (R&D, 6514-SL-050), SPON1 (R&D, 3135-SP-025), at a concentration of 1 ng µl^−1^ or LN511 (1 µg cm^−^^2^; BioLamina) for 4 h at 37 °C and cells were grown on the coated plates between days 22 and 28.

### Immunocytochemistry, image acquisition and quantification

Cells were fixed in 4% paraformaldehyde, washed in PBS and blocked in PBTA (PBS, 5% normal donkey serum (Jackson ImmunoResearch), 0.3% Triton X-100, and 1% BSA) for 1 h at room temperature. Primary antibodies were diluted in PBTA and incubations were carried out overnight at 4 °C. The primary antibodies used were the following: TH (1:500; Pel-Freez, P40101), Ki67 (1:1,000; Cell Signaling Technology, 9449), NR4A2 (1:250; Santa Cruz Biotechnology, sc990), bIII-tubulin (1:1,000; Promega, G7121), LMX1 (1:500; Millipore, AB10533), FOXA2 (1:200; R&D, AF2400), α-γH2AX (1:500; Millipore, 05-636; kindly provided by M. Farnebo), Click-iT EdU (Invitrogen, C10337) and Click-iT Plus TUNEL (Invitrogen, C10617). Corresponding secondary antibodies were Alexa Fluor Dyes (1:1,000; Invitrogen) and incubated at room temperature for 2 h. Cells were counterstained with DAPI (Thermo Fisher Scientific, D1306). Cells were then washed with PBS and stored at 4 °C in mounting medium (Dako).

Immunofluorescence images were captured with a confocal microscope (Zeiss, LSM980-Airy) with a ×20/0.45 NA objective. A minimum of six images per well were captured, with two technical replicates per condition in independent biological experiments. Quantifications of EdU and DAPI-positive nuclei were performed using Qupath^83^. TH^+^ neurons and TH^+^/EdU^+^ double-positive cells were manually counted in single-blinded experiments using Fiji^84^. All imaging and quantification parameters were kept consistent across conditions to ensure consistency and reproducibility. Results are presented as mean ± s.e.m. Statistical significance was assessed using two-sided Student’s *t* tests, with Bonferroni–Holm multiple testing correction when applicable.

### RNA extraction, cDNA synthesis and qPCR

RNA was extracted with the RNeasy Plus Mini Kit (Qiagen, 74043), and cDNA was synthesized using the High-Capacity RNA-to-cDNA Kit (Thermo Fisher Scientific, 4388950). cDNA from sorted cells was pre-amplified with SsoAdvanced PreAmp Supermix (Bio-Rad, 1725160). qPCR was performed using Fast SYBR Green Master Mix (Thermo Fisher Scientific, 4385614) and QuantStudio 5 real-time thermal cycler (Applied Biosystems). Data (dCt) were normalized to GAPDH. Results are presented as mean ± s.e.m. and statistical significance was assessed using two-sided Student’s *t* tests. Primer sequences are listed in Supplementary Table 3.

### FACS

Cells were dissociated with TrypLE Select at 37 °C for 10 min, followed by inactivation with defined trypsin inhibitor (Thermo Fisher Scientific, R007100). Cells were resuspended in PBS^−/−^ supplemented with 10% FBS and 10 µM of Y27632, filtered through a 40-μm strainer (BD Biosciences), and sorted at 4 °C on a SONY MA900 Multi-Application Cell Sorter using a 100-µm nozzle, 20 psi sheath pressure and acquisition rate of 1,000–1,200 events per second. Wild-type nonfluorescent cells were used for gating and viability was determined using DAPI staining.

### Single-cell lineage tracing

Single-cell lineage tracing was performed as previously described^55^. The lentiviral barcode library was kindly provided by M. Ratz. Briefly, following FACS isolation of tdTomato^+^ cells on differentiation day 14, cells were collected in PBS^−/−^ supplemented with 10% FBS and 10 µM of Y27632, centrifuged at 300*g* for 5 min, and resuspended in culture media supplemented with 10 µM (Y27632) and the lentiviral barcode library. Cells were plated at a density of 500,000 cells per cm² onto LN511-coated plates. On differentiation day 17, cells were dissociated with TrypLE Select at 37 °C for 10 min. Half of the cell population underwent FACS sorting to isolate EGFP^+^ barcode-containing cells, while the remaining half was replated for continued culture. On differentiation day 28, the remaining cells were subjected to another round of FACS sorting to isolate EGFP^+^ cells.

### scRNA-seq

scRNA-seq libraries were prepared using the 10x Genomics Chromium Single Cell 3’ Reagent Kit (v3). Sorted cell suspensions were adjusted to concentrations between 800 and 1,000 cells per μl and added to the 10x Chromium RT mix. For downstream cDNA synthesis (12 PCR cycles), library preparation and sequencing, we followed the manufacturer’s instructions (10x Genomics, Illumina). Libraries from two independent differentiations and two time points were sequenced and performed on an Illumina NovaSeq X platform using a 28-10-10-90 read configuration, with a targeted sequencing depth of approximately 50,000 reads per cell.

### Single-cell barcode tracing transcriptome data analysis

Single-cell transcriptome with TREX barcoding profile was demultiplexed by the National Genomics Infrastructure and raw data were transferred in FASTQ format. Raw data were aligned toward reference transcriptome combined by the human reference GRCh38 and previously published TREX barcode library chrH2B-EGFP-N (https://github.com/frisen-lab/TREX/tree/main/references). After alignment, barcode information (‘cloneIDs’) for each replicate sample containing both day 17 and day 28 time points were extracted using TREX workflow with default parameters for 10x Genomics outputs. Data were then processed using R Seurat toolkit (v4.3.0 and v5.1.0). Data underwent minimal filtering, and all cells with >500 nFeature_RNA were preserved for downstream analysis to prioritize the observation of barcode information. Merged data (combine replicates and time points) were log normalized with a scale factor of 10,000. All aligned genes were scaled and the top 2,000 variable features were calculated. Linear dimensionality reduction was performed using principal component analysis (PCA), followed by nonlinear dimensionality reduction using the top 30 principal components (PCs) based on the explained data variability. Data neighboring embedding and clustering used resolutions ranging from 0.5 to 1.0; the final clustering used a resolution of 1.0, with manual annotation of cluster identities based on calculated top marker genes for each cluster. Clusters 13 and 15 were removed due to low RNA counts, which are linked to poor barcode recovery, and cluster 19 was removed from further analysis due to relatively high mitochondrial gene expression. Visualization of annotated cell population and subpopulation top-expressing genes used ComplexHeatmap (v2.18.0). Relationship between day 17 and day 28 replicated barcodes were visualized using a Sankey plot with networkD3 (v0.4). Individual barcode highlighting from each progenitor cell population was plotted using scCustomize (v1.1.3).

### scRNA-seq data integration and latent space trajectory analysis

To assess hRgl1 and NbDA cell states in the context of midbrain development, these subpopulations were subset and integrated with midbrain scRNA-seq data from ref. ^4^. Both datasets were normalized using SCTransform before integration. Integration was performed with rapid PCA (rPCA) in Seurat (v5.3.0), using the 1,000 most variable features and the top 20 PCs. The integrated object was linearly reduced by PCA, with the top 30 PCs used for nonlinear dimensionality reduction through UMAP. Clustering was performed at a resolution of 0.8. Based on reported developmental trajectories in the reference dataset, clusters relevant to dopaminergic neuron development were retained for pseudotime trajectory analysis. Trajectories were inferred with Slingshot (v2.16.0), using known progenitor populations (Rgl1, ProgBP, ProgFP) as the starting point, without predefining terminal states.

### RNAscope human fetal tissue

Human fetal tissue was collected in Cambridge with the approval of the local research ethics committee (96/085) and was generously provided by R.A.B. Patients seeking abortions were asked whether they wished to donate fetal tissue for medical research and gave informed consent before donation. Tissue samples were dissected, fixed in 4% paraformaldehyde, and embedded in Tissue-Tek O.C.T. in Cambridge before being shipped to Sweden on dry ice. Upon arrival, tissue was cryosectioned (10 µM) and stored at −80 °C. RNAscope ISH was performed using the RNAscope Multiplex Fluorescent Detection Reagents Kit (v2; ACD Biotechne) according to the manufacturer’s instructions, with slight modifications during tissue pretreatment. Tissue slides were baked at 37 °C for 30 min, and protease III treatment was done at room temperature for 30 min. The remaining steps were carried out as per the manufacturer’s instructions. RNAscope probes used in this study were Hs-MSX1-C1 (ACD Biotechne, 470701), Hs-TH-C2 (ACD Biotechne, 441651) and Hs-NTN1-C3 (ACD Biotechne, 449531). Ethical approval for the use of human postmortem tissue was granted by the Swedish Ethics Committee (DNR, 2019-02048).

### Statistics and reproducibility

Representative immunostainings without quantification reflect standard observations, with results consistent across experiments but not all formally repeated for quantification. For experiments involving statistical testing, all biological replicates were included, and no data were excluded. No a priori statistical methods were used to determine sample size; sample sizes were based on prior literature and standard field practices. Treatment groups were assigned randomly, and file names were randomized to achieve single blinding during image quantification.

### Reporting summary

Further information on research design is available in the Nature Portfolio Reporting Summary linked to this article.

## Online content

Any methods, additional references, Nature Portfolio reporting summaries, source data, extended data, supplementary information, acknowledgements, peer review information; details of author contributions and competing interests; and statements of data and code availability are available at 10.1038/s41593-026-02200-8.

## Supplementary information

Supplementary Information Supplementary Tables 1–3.

Reporting Summary

Supplementary Data 1 BMAL1 western blot uncropped.

Supplementary Data 2 β-Aactin western blot uncropped.

Supplementary Data 3 BMAL1 plasmid sequencing result.

## Source data

Source Data Fig. 1 Statistical source data.

Source Data Fig. 2 Statistical source data.

Source Data Fig. 3 Statistical source data.

Source Data Fig. 4 Statistical source data.

Source Data Fig. 5 Statistical source data.

Source Data Fig. 6 Statistical source data.

Source Data Extended Data Fig. 1 Statistical source data.

Source Data Extended Data Fig. 6 Statistical source data.

Source Data Extended Data Fig. 7 Statistical source data.

Source Data Extended Data Fig. 8 Statistical source data.

## Data Availability

All transcriptomic data generated in this study are available in the NCBI Gene Expression Omnibus under accessions GSE82099 and GSE117394. Single-cell lineage tracing data are available in the European Genome-phenome archive under accession EGAD50000001592. Source data are provided with this paper.

## Code availability

Code used for the analysis is available at https://github.com/lamanno-epfl/rgl3_signaling_dopaminergic_analysis and https://github.com/TSun-tech/Asgrimsdottir_etal_2025.
