# Supplementary material for: Distinct radial glia subtypes regulate midbrain dopaminergic neuron development
Source: Nat Neurosci. 2026 Feb 16;29(4):810–24. doi: 10.1038/s41593-026-02200-8 (PMC13061605; doi:10.1038/s41593-026-02200-8)
Supplement: Supplementary file 1 — Supplementary Tables 1–3. [file 41593_2026_2200_MOESM1_ESM.pdf]

nature neuroscience

Supplementary information https://doi.org/10.1038/s41593-026-02200-8

Distinct radial glia subtypes regulate midbrain dopaminergic neuron development

In the format provided by the authors and unedited

SUPPLEMENTARY TABLES

Module	Number VM DEG	Module size	q-value	Enrichment Score
Black	37	551	9.12e-02	-0.55
Brown	46	856	1.08e-01	-0.14
Cyan	6	281	9.15e-04	-5.68
Green	119	2839	9.26e-03	6.10
Green Yellow	10	423	1.80e-04	-6.35
Grey	24	519	3.99e-02	-1.53
Grey60	55	175	1.03e-24	5.80
Light cyan	18	201	3.26e-02	-2.01
Light green	191	378	5.88e-136	824.97
Light yellow	2	100	2.46e-02	-3.28
Magenta	69	3087	3.56e-17	13.90
Salmon	54	287	6.10e-14	2.63
Tan	11	371	2.63e-03	-4.27

Supplementary Table 1: Summary of WGCNA analysis.

Gene	Day	Comparison	Dox	p-value
WNT1	2	shCTRL vs shBMAL1	n/a	0,0005
WNT1	4	shCTRL vs shBMAL1	n/a	0,0002
WNT1	6	shCTRL vs shBMAL1	n/a	0,00003
WNT1	8	shCTRL vs shBMAL1	n/a	0,021
NEUROG2	6	shCTRL vs shBMAL1	n/a	0,038
NEUROG2	8	shCTRL vs shBMAL1	n/a	0,009
ASCL1	6	shCTRL vs shBMAL1	n/a	0,040
ASCL1	8	shCTRL vs shBMAL1	n/a	0,034
WNT1	4	CTRL vs BMAL1-OE	2	0,016
NEUROG2	4	CTRL vs BMAL1-OE	2	0,004
ASCL1	6	CTRL vs BMAL1-OE	4	0,023
ASCL1	8	CTRL vs BMAL1-OE	4	0,002
WNT1	8	CTRL vs BMAL1-OE	6	0,004
ASCL1	6	CTRL vs BMAL1-OE	6	0,021
ASCL1	8	CTRL vs BMAL1-OE	6	0,033

Supplementary Table 2: Summary of significant p-values determined by two-tailed Student's t-test for BMAL1 qPCR analysis (Fig.5l-o).

Gene	Fwd primer	Rev primer
BMAL1	GCTCAGGAGAACCCAGGTTATC	GCATCTGCTTCCAAGAGGCTCA
EGFP	AGAACGGCATCAAGGTGAAC	TGCTCAGGTAGTGGTTGTCG
WNT1	CTTCGGCAAGATCGTCAACC	GCGAAGATGAACGCTGTTTCT
NEUROG2	GCTGGGTCTGGTACACGATT	GGCCTTCAGTCTACGGGTCT
ASCL1	ATGACGGCCATGTTGTTGTC	AAAGATGCAGGTTGTGCGATCA
MSX1	CGAGTTAAAGATGGGGAAACTG	CGAGTTAAAGATGGGGAAACTG
tdTomato	CCTGTTCCTGGGGCATGG	ATGACGGCCATGTTGTTGTC

Supplementary Table 3: Primer sequences used in study.
