# Supplementary material for: Distinct radial glia subtypes regulate midbrain dopaminergic neuron development
Source: Nat Neurosci. 2026 Feb 16;29(4):810–24. doi: 10.1038/s41593-026-02200-8 (PMC13061605; doi:10.1038/s41593-026-02200-8)
Supplement: Supplementary file 2 — Reporting Summary [file 41593_2026_2200_MOESM2_ESM.pdf]

nature portfolio

Corresponding author(s): Emilia Sif Asgrimsdottir & Goncalo Castelo-Branco

Last updated by author(s): Oct 28, 2025

Reporting Summary

Nature Portfolio wishes to improve the reproducibility of the work that we publish. This form provides structure for consistency and transparency in reporting. For further information on Nature Portfolio policies, see our Editorial Policies and the Editorial Policy Checklist.

Statistics

For all statistical analyses, confirm that the following items are present in the figure legend, table legend, main text, or Methods section.

n/a	Confirmed	
☐	☒	The exact sample size (*n*) for each experimental group/condition, given as a discrete number and unit of measurement
☐	☒	A statement on whether measurements were taken from distinct samples or whether the same sample was measured repeatedly
☐	☒	The statistical test(s) used AND whether they are one- or two-sided<br>*Only common tests should be described solely by name; describe more complex techniques in the Methods section.*
☒	☐	A description of all covariates tested
☐	☒	A description of any assumptions or corrections, such as tests of normality and adjustment for multiple comparisons
☐	☒	A full description of the statistical parameters including central tendency (e.g. means) or other basic estimates (e.g. regression coefficient) AND variation (e.g. standard deviation) or associated estimates of uncertainty (e.g. confidence intervals)
☐	☒	For null hypothesis testing, the test statistic (e.g. *F*, *t*, *r*) with confidence intervals, effect sizes, degrees of freedom and *P* value noted<br>*Give P values as exact values whenever suitable.*
☒	☐	For Bayesian analysis, information on the choice of priors and Markov chain Monte Carlo settings
☒	☐	For hierarchical and complex designs, identification of the appropriate level for tests and full reporting of outcomes
☐	☒	Estimates of effect sizes (e.g. Cohen's *d*, Pearson's *r*), indicating how they were calculated

Our web collection on statistics for biologists contains articles on many of the points above.

Software and code

Policy information about availability of computer code

Data collection	Microscopy images were acquired using a Zeiss LSM 980-Airy confocal with a 20x/0.45 NA objective, using Zen Blue v2.6. FACS was performed on a SONY MA9000 using standard software. Single-cell RNA-seq (10x Genomics Chromium 3' v3.1) was sequenced on a NovaSeq X with a read setup of 28-10-10-90. Transcriptomic reads were aligned to GRCh38, and barcode reads were aligned to a custom TREX barcode library (chrH2B-EGFP-N), targeting ~50,000 reads per cell. Bulk RNA-seq was performed on a HiSeq 2000 with 50 bp single-end reads, averaging ~9.2 million reads per sample, aligned to mm10 using Bowtie. qPCR was performed using SYBR Green on QuantStudio with 40 cycles.
Data analysis	Image quantification was performed using Fiji v2.14 (manual) and QuPath v0.4.3 (automated). Bulk and mouse single-cell RNA-seq were analyzed in R v3.4.0 using dplyr v0.5.0, reshape2 v1.4.2, FactoMineR v1.35, vegan v2.4-2, ClusterProfiler v3.4.5, ggplot2 v2.2.1, and pheatmap v1.0.8. Single-cell lineage tracing data were analyzed in Python v3.10.9 with Seurat v4.3.0 and v5.1.0; code is available at https://github.com/TSun-tech/Asgrimsdottir_etal_2015. Differential expression in bulk RNA-seq was performed using Qlucore v3.1. Cytoscape v3.3.0 and iRegulon v1.3 were used for transcription factor network analysis. GSEA v2.2.2 was used for enrichment analysis of human single-cell RNA-seq. CellChat analysis code is available at https://github.com/lamanno-epfl/rgl3_signaling_dopaminergic_analysis

For manuscripts utilizing custom algorithms or software that are central to the research but not yet described in published literature, software must be made available to editors and reviewers. We strongly encourage code deposition in a community repository (e.g. GitHub). See the Nature Portfolio guidelines for submitting code & software for further information.

Data

Policy information about availability of data

All manuscripts must include a data availability statement. This statement should provide the following information, where applicable:

- Accession codes, unique identifiers, or web links for publicly available datasets
- A description of any restrictions on data availability
- For clinical datasets or third party data, please ensure that the statement adheres to our policy

All transcriptomic data generated in this study have been deposited in the NCBI Gene Expression Omnibus under accession numbers GSE82099 and GSE117394. Single-cell lineage tracing data was deposited to the European Genome-phenome archive under the accession number EGAD50000001592. Source data are provided with this paper.

Research involving human participants, their data, or biological material

Policy information about studies with human participants or human data. See also policy information about sex, gender (identity/presentation), and sexual orientation and race, ethnicity and racism.

Reporting on sex and gender	Not determined for human fetal tissue or human embryonic stem cells.
Reporting on race, ethnicity, or other socially relevant groupings	Not determined for human fetal tissue or human embryonic stem cells.
Population characteristics	Not determined for human fetal tissue or human embryonic stem cells.
Recruitment	Human fetal tissue was collected in Cambridge. Patients seeking abortions were asked about their interest to donate tissue for medical research and gave informed consent prior to donation.
Ethics oversight	Collection of tissue was approved by the local ethics committee (96/085). Ethical approval for the use of human post-mortem tissue was granted by the Swedish Ethics Committee (DNR: 2019-02048).

Note that full information on the approval of the study protocol must also be provided in the manuscript.

Field-specific reporting

Please select the one below that is the best fit for your research. If you are not sure, read the appropriate sections before making your selection.

☒ Life sciences ☐ Behavioural & social sciences ☐ Ecological, evolutionary & environmental sciences

For a reference copy of the document with all sections, see nature.com/documents/nr-reporting-summary-flat.pdf

Life sciences study design

All studies must disclose on these points even when the disclosure is negative.

Sample size	No a priori statistical methods were used to determine sample size. Sample sizes (n = 3–6) were based on prior literature and standard practices in the field. For imaging quantification,6 20x fields were randomly selected from 2 wells per condition, using a minimum of 3 biological replicates. For single-cell lineage tracing, two biological replicates were used, which is consistent with accepted standards and supported by high reproducibility between replicates.
Data exclusions	No samples were excluded from the analysis. For single-cell lineage tracing data, minimal filtering was applied during quality control. Cells with fewer than 500 detected genes (nFeature_RNA) were excluded to ensure sufficient transcriptomic information.
Replication	Ligand, ECM treatment, and BMAL1 experiments were independently repeated more than three times, with all biological replicates supporting the main findings. Single-cell lineage tracing was performed with two biological replicates, which was justified by the low variability observed between samples.
Randomization	Treatment groups were assigned randomly. File names were randomized to blind the experimenter during imaging quantification and cell counting.
Blinding	Cell counting was performed in single-blinded experiments by two independent experimenters, each blinded to both the experimental condition and treatment group. Each experimenter analyzed a separate set of samples.

Reporting for specific materials, systems and methods

We require information from authors about some types of materials, experimental systems and methods used in many studies. Here, indicate whether each material, system or method listed is relevant to your study. If you are not sure if a list item applies to your research, read the appropriate section before selecting a response.

Materials & experimental systems

n/a	Involved in the study
☐	☒ Antibodies
☐	☒ Eukaryotic cell lines
☒	☐ Palaeontology and archaeology
☐	☒ Animals and other organisms
☒	☐ Clinical data
☒	☐ Dual use research of concern
☒	☐ Plants

Methods

n/a	Involved in the study
☒	☐ ChIP-seq
☐	☒ Flow cytometry
☒	☐ MRI-based neuroimaging

Antibodies

Antibodies used	TH (1:1000, Pel-Freez, P40101), Ki67 (1:500, Cell Signaling Technology, 9449), NR4A2/Nurr1 (1:500, Santa Cruz, sc990), βIII-tubulin (1:500, Promega, G7121), LMX1 (1:1000, Millipore, AB10533), FOXA2 (1:1000, R&D Systems, AF2400), BMAL1 (1:1000,Abcam, ab93806), γH2AX (1:500, Millipore, 05-636), and β-ACTIN (1:500, Abcam, ab6276). Secondary antibodies were AlexaFluor from Invitrogen.
Validation	All antibodies used in this study are widely used in the field and have been validated by the respective suppliers. They have been cited in prior publications, and have also been successfully and routinly used in previous studies by our group.

Eukaryotic cell lines

Policy information about cell lines and Sex and Gender in Research

Cell line source(s)	WA09 hESCs were obtained from WiCell. HS980 (Rodin et al., 2014) and Sai2 (Tailor et al., 2013) hLT-NES cells were kindly provided by Prof. Fredrik Lanner and Prof. Anna Falk, respectively.
Authentication	WA09 cells were authenticated by WiCell. HS980 (WT) and Sai2 cell lines were previously authenticated and described in Rodin et al. (2014) and Tailor et al. (2013),respectively. The transgenic HS980 (MSX1-tdTom) line was authenticated as described in Asgrimsdottir et al., 2024. No additional authentication was performed by the authors.
Mycoplasma contamination	All cell lines used in this study were regularly tested and confirmed to be free of mycoplasma contamination.
Commonly misidentified lines (See ICLAC register)	None of the cell lines used in this study are known to be misidentified or listed in ICLAC or Cellosaurus databases.

Animals and other research organisms

Policy information about studies involving animals; ARRIVE guidelines recommended for reporting animal research, and Sex and Gender in Research

Laboratory animals	Transgenic TH-GFP mice [Tg(Th-EGFP)DJ76Gsat], where EGFP is expressed under the control of the TH promoter, were used in this study. This line was originally described by Matsushita et al., 2002, and is maintained on a (C57BL/6J × DBA/2J)F2 genetic background.
Wild animals	No wild animals were used in the study.
Reporting on sex	Gender was not determined for the mouse embryos.
Field-collected samples	No field collected samples were used in the study.
Ethics oversight	Ethical approval for mice experimentation was granted by the local ethics committee, Stockholm Norra Djurförsöksetiska Nämnd number N326/12 and N158/15.

Note that full information on the approval of the study protocol must also be provided in the manuscript.

Plants

Seed stocks

Report on the source of all seed stocks or other plant material used. If applicable, state the seed stock centre and catalogue number. If plant specimens were collected from the field, describe the collection location, date and sampling procedures.

Novel plant genotypes

Describe the methods by which all novel plant genotypes were produced. This includes those generated by transgenic approaches, gene editing, chemical/radiation-based mutagenesis and hybridization. For transgenic lines, describe the transformation method, the number of independent lines analyzed and the generation upon which experiments were performed. For gene-edited lines, describe the editor used, the endogenous sequence targeted for editing, the targeting guide RNA sequence (if applicable) and how the editor was applied.

Authentication

Describe any authentication procedures for each seed stock used or novel genotype generated. Describe any experiments used to assess the effect of a mutation and, where applicable, how potential secondary effects (e.g. second site T-DNA insertions, mosiacism, off-target gene editing) were examined.

Flow Cytometry

Plots

Confirm that:

☒ The axis labels state the marker and fluorochrome used (e.g. CD4-FITC).

☒ The axis scales are clearly visible. Include numbers along axes only for bottom left plot of group (a 'group' is an analysis of identical markers).

☒ All plots are contour plots with outliers or pseudocolor plots.

☒ A numerical value for number of cells or percentage (with statistics) is provided.

Methodology

Sample preparation

Cells were dissociated into single-cell suspensions using TrypLE Select, and passed through a 40 µm filter. They were then resuspended in ice-cold PBS containing 10% FBS and 10 µM Y27632, and kept on ice to preserve viability.

Instrument

Sony MA900

Software

Sony MA9000 standard operating software

Cell population abundance

MSX1-tdTomato+ cells were 7.98% at day 14 (n = 3), gated using WT HS980 controls. EGFP+ cells were 14.31% at day 17 and 22.01% at day 28 (n = 2), gated using non-transduced controls.

Gating strategy

WT HS980 cells were used to gate tdTomato-positive populations. Untransduced tdTomato-MSX1 HS980 cells were used to gate GFP-positive populations.

☒ Tick this box to confirm that a figure exemplifying the gating strategy is provided in the Supplementary Information.
