# Supplementary figures and images for: Distinct radial glia subtypes regulate midbrain dopaminergic neuron development
Source: Nat Neurosci. 2026 Feb 16;29(4):810–24. doi: 10.1038/s41593-026-02200-8 (PMC13061605; doi:10.1038/s41593-026-02200-8)

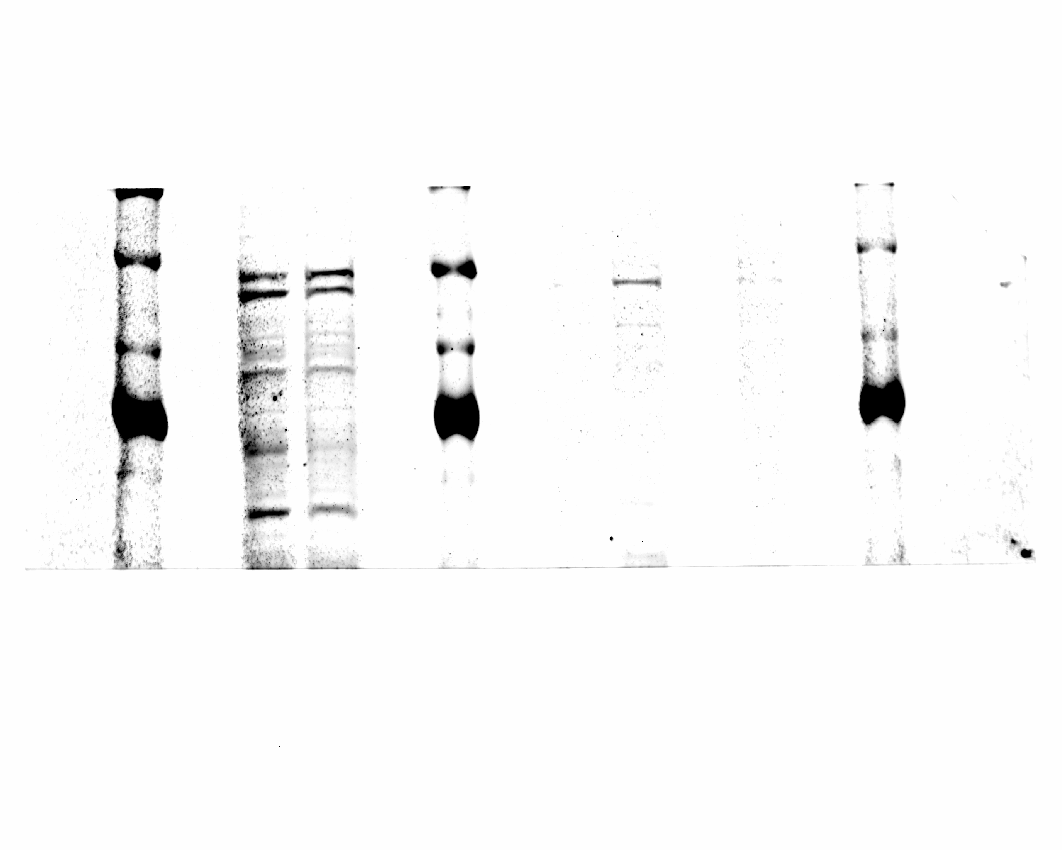

Supplement: Supplementary file 3 — BMAL1 western blot uncropped. [file 41593_2026_2200_MOESM3_ESM.tif]

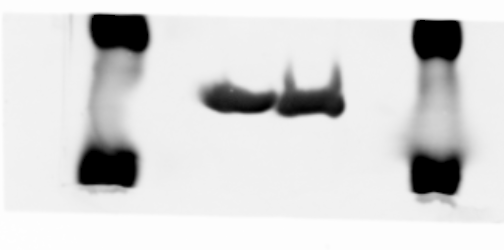

Supplement: Supplementary file 4 — β-Aactin western blot uncropped. [file 41593_2026_2200_MOESM4_ESM.tif]
