# Supplementary material for: Distinct radial glia subtypes regulate midbrain dopaminergic neuron development
Source: Nat Neurosci. 2026 Feb 16;29(4):810–24. doi: 10.1038/s41593-026-02200-8 (PMC13061605; doi:10.1038/s41593-026-02200-8)

Clip. 1 BQ 20 WL 10

Sequence: ET_Antisense_Arntl_premix

Clipped length:	983	Samples:	13055	Quality: 0 - 9	
Left clip:	10	Bases:	1096	10 - 19	
Right clip:	992	Average spacing:	12.0	20 - 29	
Avg. qual. in clip.:	53.38	Average quality >=	10: 50, 20: 50, 30: 975	>= 30	

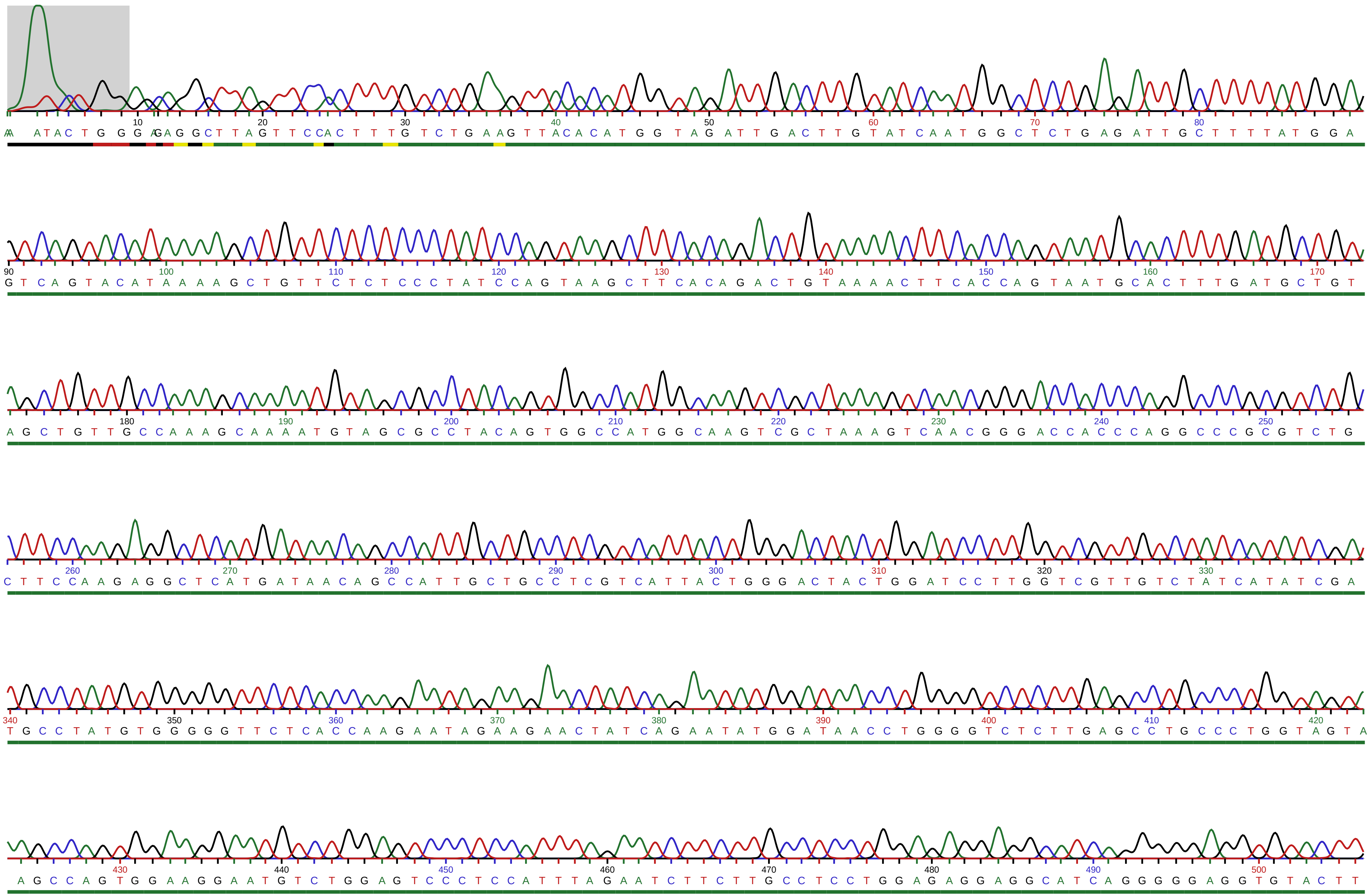

Clip. 1 BQ 20 WL 10 Sequence: ET_Antisense_Arntl_premix

Clipped length:	983	Samples:	13055	Quality:	0 - 9	
Left clip:	10	Bases:	1096		10 - 19	
Right clip:	992	Average spacing:	12.0		20 - 29	
Avg. qual. in clip.:	53.38	Average quality >=	10: 50, 20: 50, 30: 975		>= 30	

06.12.2016

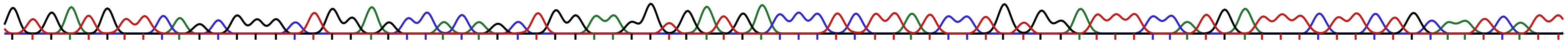

510 520 530 540 550 560 570 580 590

G T G A T G T T C A G C G G G C T G G A G C C A C A G C T G G A A G G T G A T G A C C C T C T T A T C C T G T G G A T T T C C A T G A T T T C T T C T G C A A T C A T T

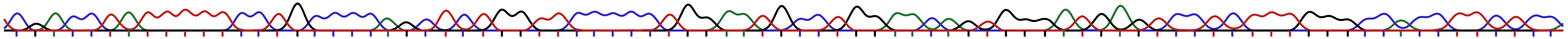

600 610 620 630 640 650 660 670

C G A C C T A T T T T T C C T G C C C C A G C T C T G G T T C C C C C T G G A A T G C C T G G A A C A G T G G G A T G A G T C C T C T T T G G G C C A C C T T C T C C

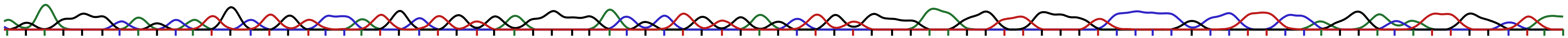

680 690 700 710 720 730 740 750

A G A G G G C A G C A T G C T G T C C A T G C T G T G A G G G G A C G C T G T G A G C T G T G G G A A G G T T G G G T C C C C G C C T T C C A G G A C A T T G G C T A A

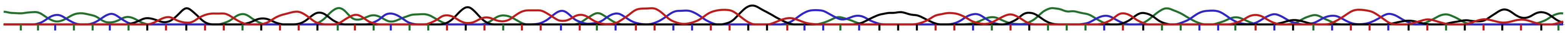

760 770 780 790 800 810 820 830 840

A A C A A C A G T G T T A G T T G A T A C A A T G T A T T C T A C T T C C T T G G T C C A C G G G T T C A T G A A A C T G A A C C A T C G A C T T C G T A G T G T G A

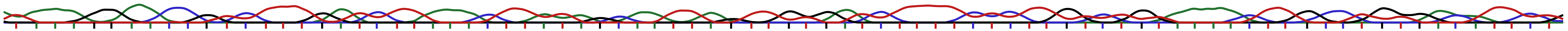

850 860 870 880 890 900 910 920

T A A A G G A A C C G T C T T T G A T C T T A A A C T T A T A G C A A T T A G T T G T G A T C T T T T C T C T T G T C T G T A A A A C T T G C C T G T G A C A T T C T

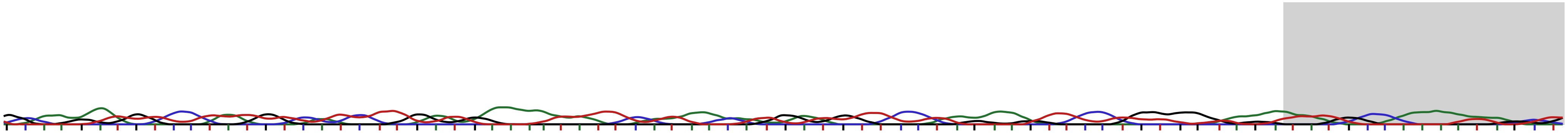

930 940 950 960 970 980 990 1000

G C A A G A T G T C C T A T G T C A T C T T G A T G A A A A T A T T C A T A A C A T G A T G T T C C T A G A A G T T C C T G T G G T A G A T A T G C C A A A T A G C T

Clip. 1 BQ 20 WL 10

Sequence: ET_Antisense_Arntl_premix

Clipped length:	983	Samples:	13055	Quality:	0 - 9
Left clip:	10	Bases:	1096		10 - 19
Right clip:	992	Average spacing:	12.0		20 - 29
Avg. qual. in clip.:	53.38	Average quality >=	10: 50, 20: 50, 30: 975		>= 30

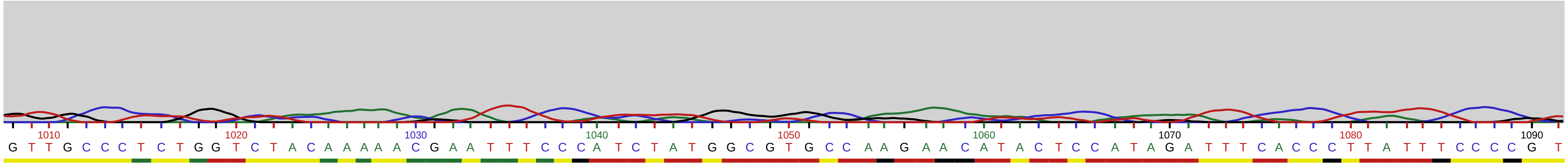

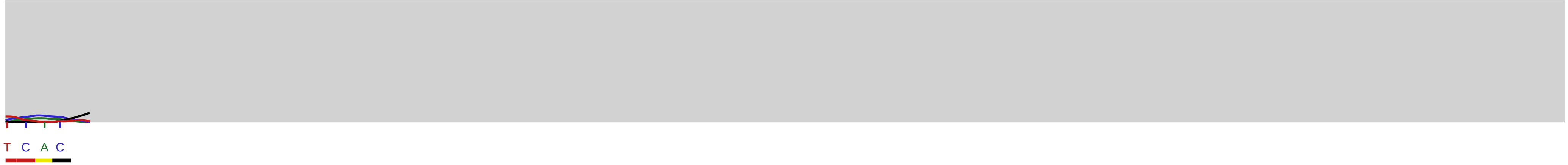

Supplement: Supplementary file 5 — BMAL1 plasmid sequencing result. [file 41593_2026_2200_MOESM5_ESM.pdf]
